# Supplementary material for: Early interventions for post-traumatic stress following musculoskeletal trauma: protocol for a systematic review and meta-analysis
Source: BMJ Open. 2022 Sep 23;12(9):e065590. doi: 10.1136/bmjopen-2022-065590 (PMC9511568; doi:10.1136/bmjopen-2022-065590)
Supplement: Supplementary data [file bmjopen-2022-065590supp002.pdf]

Early interventions for post-traumatic stress following musculoskeletal trauma: Protocol for a systematic review and meta-analysis

Medline search strategy

1. Post-traumatic stress symptom

Post-traumatic stress.mp **OR** Post-traumatic stress disorder.mp **OR** Post traumatic.mp **OR** Post-traumatic stress symptom.mp **OR** Stress symptoms.mp **OR** Post-trauma*.mp **OR** PTSS.mp or PTSD.mp **OR** Acute stress disorder.mp **OR** ASD.mp

2. Musculoskeletal trauma

Musculoskeletal trauma.mp **OR** musculoskeletal injury.mp **OR** Orthopaedic trauma.mp **OR** Orthopaedic injuries.mp **OR** Orthopedic trauma.mp **OR** Orthopedic injury.mp **OR** Musculoskeletal.mp **OR** Musculoskeletal inj* **OR** Lower extremity inj* **OR** Upper extremity inj* **OR** Exp fractures, bone **OR** Exp whiplash injuries **OR** Whiplash.mp **OR** Athletic injuries.mp **OR** Traumatic injuries.mp **OR** Falls.mp **OR** Traffic collision.mp **OR** Exp multiple trauma **OR** Traumatic inj* **OR** Gunshot injury* **OR** Stab wound*

3. Intervention

Psychotherapies.mp **OR** psychotherap* **OR** physiotherap* **OR** physical therap* **OR** emdr.mp **OR** eye movement desensiti* **OR** prevention.mp **OR** intervention.mp **OR** therap* **OR** psychoeducation.mp **OR** treatment.mp **OR** cognitive behavioural therapy.mp **OR** CBT.mp **OR** rehabilitation.mp **OR** imagery.mp **OR** exercis*.mp **OR** movement therapy.mp **OR** exercise training.mp **OR** physical activit*.mp **OR** aerobic exercise.mp **OR** physical exercise.mp **OR** resistance training.mp **OR** strength training.mp **OR** endurance exercise.mp **OR** physical therap*.mp **OR** manual therap*.mp **OR** massage.mp **OR** acupuncture **OR** aerobic.mp

Jadhakhan F, *et al. BMJ Open* 2022; 12:e065590. doi: 10.1136/bmjopen-2022-065590

CINAHL search strategy

1. **Post-traumatic stress symptom**

Post traumatic **OR** Post-traumatic stress symptom **OR** Post-trauma **OR** PTSS **OR** Post-traumatic stress **OR** Post-traumatic stress disorder **OR** Post-traumatic stress symptom **OR** PTSD **OR** Acute stress disorder **OR** ASD

2. **Musculoskeletal trauma**

Musculoskeletal trauma **OR** Musculoskeletal injury **OR** Orthopaedic trauma **OR** Orthopaedic injury **OR** Orthopedic trauma **OR** Lower extremity injury **OR** Upper extremity injury **OR** Fractures, bone **OR** Whiplash injuries **OR** Whiplash **OR** Athletic injury **OR** Multiple trauma **OR** Traumatic injury **OR** Traumatic injuries **OR** Falls **OR** Gunshot injury **OR** Stab wound **OR** Traffic collision

3. **Intervention**

Psychotherapies **OR** psychotherapy **OR** physiotherapy **OR** physical therapy **OR** EMDR **OR** eye movement desensitisation **OR** prevention **OR** intervention **OR** therapy **OR** psychoeducation **OR** treatment **OR** cognitive behavioural therapy **OR** CBT **OR** rehabilitation **OR** imagery **OR** exercise **OR** movement therapy **OR** exercise training **OR** physical activity **OR** aerobic exercise **OR** physical exercise **OR** resistance training **OR** strength training OR endurance exercise **OR** physical therapy **OR** manual therapy **OR** massage **OR** acupuncture **OR** aerobic

Jadhakhan F, *et al. BMJ Open* 2022; 12:e065590. doi: 10.1136/bmjopen-2022-065590

EMBASE search strategy

1. **Post-traumatic stress symptom**

Post traumatic.ab, ti **OR** Post-traumatic stress symptom$.ab, ti **OR** Post-trauma.mp **OR** PTSS.mp **OR** Post traumatic stress.mp **OR** Post-traumatic stress disorder.mp **OR** Post traumatic.mp **OR** Post-traumatic stress symptom.mp **OR** PTSD.mp **OR** Acute stress disorder.mp **OR** ASD.mp

PTSS.tw

2. **Musculoskeletal trauma**

Musculoskeletal trauma.mp **OR** musculoskeletal injury.mp **OR** Orthopaedic trauma.mp **OR** orthopedic trauma.mp **OR** Fractures, bone.mp **OR** Whiplash injuries.mp **OR** Whiplash.mp **OR** Multiple trauma.mp **OR** Traumatic injuries.mp **OR** Falls.mp **OR** Gunshot injury.mp **OR** Stab wound.mp **OR** Traffic collision.mp **OR** Musculoskeletal inj$.ab,ti **OR** Orthopaedic inj$.ab,ti **OR** Orthopedic inj$.ab,ti **OR** Lower extremity inj$.ab,ti **OR** Upper extremity inj$.ab,ti **OR** Athletic inj$.ab,ti **OR** Traumatic inj$.ab,ti

3. **Intervention**

Psychotherap$.ab,ti **OR** Psychotherapies.mp **OR** Psychotherapy.mp **OR** physiotherapy$.ab,ti **OR** Physiotherapies.mp **OR** Physiotherapy.mp **OR** physical therap$.ti,ab **OR** physical therapies.mp **OR** physical therapy.mp **OR** EMDR.mp **OR** eye movement desensitisation.mp **OR** desensitization.mp **OR** prevention.mp **OR** intervention.mp **OR** therap$.ab,ti **OR** therapies.mp **OR** therapy.mp **OR** psychoeducation.mp **OR** treatment.mp **OR** cognitive behavioural therapy.mp **OR** CBT.mp **OR** rehabilitation.mp **OR** imagery.mp OR exercise.mp **OR** movement therapy.mp **OR** exercise training.mp **OR** physical activity.mp **OR** aerobic exercise.mp **OR** physical exercise.mp **OR** resistance training.mp **OR** strength training.mp **OR** endurance exercise.mp

Jadhakhan F, *et al. BMJ Open* 2022; 12:e065590. doi: 10.1136/bmjopen-2022-065590

OR physical therapy.mp **OR** manual therapy.mp **OR** massage.mp **OR** acupuncture **OR** aerobic.mp

PsycINFO search strategy

1. **Post-traumatic stress symptom**

Post-traumatic stress disorder.mp **OR** post-traumatic stress disorders.mp **OR** posttraumatic stress disorder.mp **OR** posttraumatic stress disorders.mp **OR** disorder* **OR** post-traumatic.mp **OR** post-traumatic stress symptom*.mp **OR** post traumatic stress symptom*.mp **OR** PTSS.mp **OR** PTSD.mp **OR** acute stress disorder.mp **OR** ASD.mp

2. **Musculoskeletal trauma**

Musculoskeletal trauma.mp **OR** musculoskeletal injur*.mp **OR** orthopaedic trauma.mp **OR** orthopaedic injur*.mp **OR** orthopedic trauma.mp **OR** lower extremity injur*.mp **OR** upper extremity injur*.mp **OR** fracture*.mp **OR** bone.mp **OR** whiplash injur*.mp **OR** whiplash **OR** athletic injur*.mp **OR** multiple trauma.mp **OR** traumatic injur*.mp **OR** traumatic injur*.mp **OR** falls.mp **OR** gunshot injur*.mp **OR** stab wound*.mp **OR** traffic collision*.mp

3. **Intervention**

Psychotherap* **OR** Psychotherapies.mp **OR** Psychotherapy.mp **OR** physiotherapy* **OR** Physiotherapies.mp **OR** Physiotherapy.mp **OR** physical therap* **OR** physical therapies.mp **OR** physical therapy.mp **OR** EMDR.mp **OR** eye movement desensitisation.mp **OR** desensitization.mp **OR** prevention.mp **OR** intervention.mp **OR** therap* **OR** therapies.mp **OR** therapy.mp **OR** psychoeducation.mp **OR** treatment.mp **OR** cognitive behavioural therapy.mp **OR** CBT.mp **OR** rehabilitation.mp **OR** imagery.mp OR exercis* OR movement therapy.mp OR exercise training.mp OR physical activit* OR aerobic exercise.mp OR physical exercise.mp OR resistance training.mp OR strength training.mp OR endurance exercise.mp **OR** physical therap* OR manual therap* OR massage.mp OR acupuncture OR aerobic.mp

Jadhakhan F, *et al. BMJ Open* 2022; 12:e065590. doi: 10.1136/bmjopen-2022-065590

PubMed search strategy

1. **Post-traumatic stress symptom**

Post-traumatic stress **OR** Post-traumatic stress disorder **OR** Post traumatic **OR** Post-traumatic stress symptom **OR** Stress symptoms **OR** Post-trauma **OR** PTSS **OR** PTSD **OR** Acute stress disorder **OR** ASD

2. **Musculoskeletal trauma**

Musculoskeletal trauma **OR** musculoskeletal injury **OR** Orthopaedic trauma **OR** Orthopaedic injuries **OR** Orthopedic trauma **OR** Orthopedic injury **OR** Musculoskeletal **OR** Orthopaedic **OR** Musculoskeletal injury **OR** Lower extremity injury **OR** Upper extremity injury **OR** fractures **OR** bone **OR** whiplash injuries **OR** Whiplash **OR** Athletic injuries **OR** Traumatic injuries **OR** Falls **OR** Traffic collision **OR** multiple trauma **OR** Traumatic injury **OR** Gunshot injury OR Stab wound

3. **Intervention**

Psychotherapy **OR** Psychotherapies **OR** Physiotherapy **OR** Physiotherapies **OR** physical therapy **OR** physical therapies **OR** EMDR **OR** eye movement desensitisation **OR** desensitization **OR** prevention **OR** intervention **OR** therapy **OR** therapies **OR** psychoeducation **OR** treatment **OR** cognitive behavioural therapy **OR** CBT **OR** rehabilitation **OR** imagery **OR** exercise **OR** movement therapy **OR** exercise training **OR** physical activity **OR** aerobic exercise **OR** physical exercise **OR** resistance training **OR** strength training OR endurance exercise **OR** physical therapy **OR** manual therapy **OR** massage **OR** acupuncture **OR** aerobic

Jadhakhan F, *et al. BMJ Open* 2022; 12:e065590. doi: 10.1136/bmjopen-2022-065590

Web of Science search strategy

1. **Part 1: Post-traumatic stress symptom**

Post-traumatic stress **OR** Post-traumatic stress disorder **OR** Post traumatic **OR** Post-traumatic stress symptom **OR** Stress symptoms **OR** Post-trauma **OR** PTSS **OR** PTSD **OR** Acute stress disorder **OR** ASD

2. **Musculoskeletal trauma**

Musculoskeletal trauma **OR** musculoskeletal injury **OR** Orthopaedic trauma **OR** Orthopaedic injuries **OR** Orthopedic trauma **OR** Orthopedic injury **OR** Musculoskeletal **OR** Orthopaedic **OR** Musculoskeletal injury **OR** Lower extremity injury **OR** Upper extremity injury **OR** fractures **OR** bone **OR** whiplash injuries **OR** Whiplash **OR** Athletic injuries **OR** Traumatic injuries **OR** Falls **OR** Traffic collision **OR** multiple trauma **OR** Traumatic injury **OR** Gunshot injury OR Stab wound

3. **Intervention**

Psychotherapy **OR** Psychotherapies **OR** Physiotherapy **OR** Physiotherapies **OR** physical therapy **OR** physical therapies **OR** EMDR **OR** eye movement desensitisation **OR** desensitization **OR** prevention **OR** intervention **OR** therapy **OR** therapies **OR** psychoeducation **OR** treatment **OR** cognitive behavioural therapy **OR** CBT **OR** rehabilitation **OR** imagery **OR** exercise **OR** movement therapy **OR** exercise training **OR** physical activity **OR** aerobic exercise **OR** physical exercise **OR** resistance training **OR** strength training **OR** endurance exercise **OR** physical therapy **OR** manual therapy **OR** massage **OR** acupuncture **OR** aerobic

Jadhakhan F, *et al. BMJ Open* 2022; 12:e065590. doi: 10.1136/bmjopen-2022-065590
