# Supplementary material for: Early interventions for post-traumatic stress following musculoskeletal trauma: protocol for a systematic review and meta-analysis
Source: BMJ Open. 2022 Sep 23;12(9):e065590. doi: 10.1136/bmjopen-2022-065590 (PMC9511568; doi:10.1136/bmjopen-2022-065590)
Supplement: Author's manuscript [file bmjopen-2022-065590.draft_revisions.pdf]

BMJ Open

BMJ Open is committed to open peer review. As part of this commitment we make the peer review history of every article we publish publicly available.

When an article is published we post the peer reviewers' comments and the authors' responses online. We also post the versions of the paper that were used during peer review. These are the versions that the peer review comments apply to.

The versions of the paper that follow are the versions that were submitted during the peer review process. They are not the versions of record or the final published versions. They should not be cited or distributed as the published version of this manuscript.

BMJ Open is an open access journal and the full, final, typeset and author-corrected version of record of the manuscript is available on our site with no access controls, subscription charges or pay-per-view fees (http://bmjopen.bmj.com).

If you have any questions on BMJ Open's open peer review process please email info.bmjopen@bmj.com

Early interventions for post-traumatic stress following musculoskeletal trauma: Protocol for a systematic review and meta-analysis

Journal:	*BMJ Open*
Manuscript ID	bmjopen-2022-065590
Article Type:	Protocol
Date Submitted by the Author:	13-Jun-2022
Complete List of Authors:	Jadhakhan, Ferozkhan; University of Birmingham, School of Sport, Exercise and Rehabilitation Sciences<br>Evans, David; University of Birmingham, School of Sport, Exercise and Rehabilitation Sciences<br>Falla, Deborah; University of Birmingham
Keywords:	Musculoskeletal disorders < ORTHOPAEDIC & TRAUMA SURGERY, TRAUMA MANAGEMENT, Rehabilitation medicine < INTERNAL MEDICINE, Trauma management < ORTHOPAEDIC & TRAUMA SURGERY

SCHOLARONE™
Manuscripts

Early interventions for post-traumatic stress following musculoskeletal trauma: Protocol for a systematic review and meta-analysis

Ferozkhan Jadhakhan[1], David W Evans[1], Deborah Falla[1*]

1. Centre of Precision Rehabilitation for Spinal Pain, School of Sport, Exercise and Rehabilitation Sciences, College of Life and Environmental Sciences, University of Birmingham, Edgbaston, Birmingham, B15 2TT, United Kingdom

Correspondence:

Professor Deborah Falla
Centre of Precision Rehabilitation for Spinal Pain
School of Sport, Exercise and Rehabilitation Sciences,
College of Life and Environmental Sciences,
University of Birmingham,
Birmingham, B15 2TT, UK.
Tel: +44 121 415 4220
Email: d.falla@bham.ac.uk

For peer review only

ABSTRACT

Introduction: Several interventions have been shown to alleviate post-traumatic stress symptoms (PTSS) but little is known about which intervention provides the greatest effect for PTSS following a traumatic event. This systematic review aims to estimate the effect of early interventions on PTSS following musculoskeletal trauma.

Methods/Analysis: Development of this systematic review protocol was guided by the Preferred Reporting Items for Systematic Reviews and Meta-Analysis-P. This review will include randomised controlled trials (RCT) and non-randomised controlled studies evaluating the effect of non-surgical and non-pharmacological interventions on PTSS in adults (aged ≥18 years). MEDLINE, PsycINFO, EMBASE, CINAHL, ZETOC, PROSPERO, Web of Science, PubMed and Google Scholar as well as key journals/grey literature will be searched from inception to 31st July 2022. Only articles published in English will be considered eligible. Two independent reviewers will search, screen studies, extract data, and assess risk of bias using the Cochrane Risk of Bias tool V2 (RoB 2) or the Risk Of Bias in Non-randomized Studies of Interventions (ROBINS-I). Mean difference or standardised mean difference will be extracted with accompanying 95% confidence intervals (CI) and p-values where this is reported. Group effect size will be extracted and reported. Symptoms of PTSS will be ascertained using standardised mean differences (continuous) and diagnosis of PTSS using risk ratio (dichotomous). If possible, study results will be pooled into a meta-analysis. A narrative synthesis of the results will be presented if heterogeneity is high. The overall quality of evidence will be assessed using the GRADE.

Ethics and dissemination: Ethical approval is not required for this systematic review since data from published studies will be used. This review is expected to provide a better understanding of the effect of early intervention for PTSS following musculoskeletal trauma. Findings of this review will be disseminated in peer-reviewed publications and through national and international conferences.

Prospero registration: CRD42022333905

Keywords: Musculoskeletal trauma, psychological intervention, post-traumatic stress symptoms, early intervention

For peer review only

INTRODUCTION

Post-traumatic stress disorder (PTSD) is triggered following exposure to a traumatic event, such as violence, disasters, serious accidents, and injury[1-2]. PTSD is characterised by four classes of symptoms, re-experiencing the trauma, avoidance of stimuli related to the event, emotional numbness, and hyper-arousal[3]. Symptoms of PTSD can have a significant impact on the day to day life of those exposed to trauma and their families[4]. On the other hand, acute stress disorder (ASD) is characterised by severe stress symptoms that may occur within the first month after a traumatic event and last for a maximum of four weeks. The disorder includes symptoms of intrusion, dissociation, negative mood, avoidance, and arousal[5]. If these symptoms persists longer than one month after the traumatic event, PTSD is diagnosed[6]. These symptoms generally affect the social, occupational, interpersonal relationship, and physical health of those exposed to a traumatic event. A precursor of PTSD is the development of post-traumatic stress symptoms (PTSS), which is defined as symptoms consistent with those of PTSD but occurs earlier (at least 30 days) after experiencing a traumatic event[7]. PTSS is now recognised as a serious health issue[8].

Musculoskeletal injuries are common and can lead to severe long-term pain and disability. They are the leading cause of severe morbidity and mortality worldwide[9]. In 2016, an estimated 146,000 deaths resulting from road traffic accidents were reported in the European Union (EU-27 countries), equivalent to 3.2% of all deaths registered for that year[10]. Globally, road traffic accidents killed approximately 1.35 million people in 2016, an estimated 3,700 deaths per day and injured more than 50 million people[11]. In England an estimated 20,000 cases of major trauma occur each year resulting in 5,400 deaths with many sustaining permanent disability needing long term care[12]. Annually in the United Kingdom (UK), between 40,000-90,000 individuals are involved in a major traumatic accident, of which 50% will have sustained a musculoskeletal injury[12-14]. Some of the most common major trauma include injuries to the head, neck, spine, chest, limbs, abdomen and pelvis. In England, the most common

For peer review only - http://bmjopen.bmj.com/site/about/guidelines.xhtml

mechanisms of injury are road traffic accidents and falls[15]. It is estimated that the annual loss in economic output as a result of major trauma is between £3.3 and £3.7 billion in the UK[12]. The annual emergency and hospital treatment for trauma care is estimated to cost the National Health Service (NHS) an estimated £300-£400 million[13].

Although PTSD and ASD has long been established as serious health concerns following a major physical trauma, PTSS is also now considered a serious health issue associated with significant distress and impairment[16,17]. PTSS impacts both quality of life and healthcare resources, which is why there is a need for providing effective and timely intervention strategies. Several interventions have been shown to be effective in the management of PTSS. These interventions are broadly psychological and include treatment approaches such as behavioural (exposure) therapy, trauma focused cognitive behavioural therapy (CBT), supportive (psychodynamic and interpersonal) psychotherapy, eye movement desensitisation and reprocessing (EMDR), brief eclectic psychotherapy, and other therapies performed individually or in groups[18]. These treatments aim at reducing PTSS by working through stressors related to the traumatic event[19]. For example, Bisson and colleagues[20] compared four 1-hour CBT sessions to no intervention (standard care only, involving no formal intervention) in individuals with evidence of PTSS. The results showed that Impact of Event Scale (IES) scores were reduced in the intervention group at 13 months compared to the control group (adjusted mean difference=8.4, 95% CI 2.4–14.36). On the other hand, a systematic review and meta-analysis of multiple psychological interventions designed to treat PTSD symptoms in military personnel within three months of a traumatic event, found little evidence to support any interventions reducing PTSD symptoms post treatment, although Trauma Focus Cognitive Behavioural Therapy (CBT-TF) was associated with a reduction in PTSD symptoms post treatment compared to waitlist-control (standardised mean difference (SMD) −1.22; 95% CI, −1.78 to −0.66)[21]. Another study[22] compared the effects of a brief CBT intervention on

For peer review only - http://bmjopen.bmj.com/site/about/guidelines.xhtml

PTSS severity in patients admitted to emergency department (ED) following motor vehicle crashes (MVA) found no differential effect on PTSS measured by the Impact of Events Scale-Revised. Many of these studies have potential methodological shortcomings, justifying a robust review of empirical evidence evaluating these psychological interventions in individuals with PTSS following musculoskeletal trauma. Furthermore, because of the wide range of available psychological interventions for PTSS, it is important to identify which intervention(s), whether alone or in combination has the greatest effect on PTSS when applied within three months of the traumatic event. To date, no systematic review has investigated the effect of early psychological interventions for PTSS following musculoskeletal trauma.

Aims

This systematic review aims to:

- Estimate the effect of early psychological interventions on PTSS, when commenced within 3 months of a traumatic event causing musculoskeletal injuries
- Estimate the effect of different types of interventions on PTSS severity

METHODS

This systematic review protocol follows the reporting guidelines according to the Preferred Reporting Items for Systematic Reviews and Meta-Analysis Protocols (PRISMA-P checklist)[23] (Supplementary File 1). This review protocol was registered with the International Prospective Register of Systematic Reviews (PROSPERO registration: CRD42022333905) on the 19th of May 2022 (https://www.crd.york.ac.uk/prospero/export_details_pdf.php). The planned start date for this review is 31st July 2022 and the planned end date is 30th September 2022.

Search strategy

The following citation databases MEDLINE, PsycINFO, EMBASE, CINAHL, ZETOC, PROSPERO, Web of Science, PubMed, Google Scholar and Cochrane central register

For peer review only - http://bmjopen.bmj.com/site/about/guidelines.xhtml

of controlled trials as well as key journals/grey literature will be searched from inception to 31st July 2022. Only articles published in English will be considered eligible. The following key words will be used to retrieve relevant articles: trauma, musculoskeletal, acute stress disorder, post-traumatic stress, ASD, PTSD, PTSS, cognitive behavioural, EMDR, psychological, therapy, psychological intervention. A search strategy for each database can be found in supplementary file 2.

Inclusion criteria

The selection criteria for inclusion/exclusion of studies will follow the Participants, Interventions, Comparators, Outcomes and Study design (PICOS) framework[24].

Population: Individuals aged ≥18 years who have sustained a physically traumatic event resulting in one or more musculoskeletal injuries. In studies with categorised aged group >90% of participants must be adults (≥18 years).

Intervention: Any non-pharmaceutical and non-surgical intervention designed to reduce symptoms of post-traumatic stress must be included. Types of intervention may include, for example: trauma focussed psychodynamic psychotherapy[25], psychoeducation[26], cognitive behavioural therapy (CBT)[27], and eye movement desensitisation and reprocessing (EMDR)[28].

Comparator(s)/control: Comparator group(s) can be: true control (i.e. no intervention provided), care as usual, pharmaceutical or surgical interventions, wait-list controls, placebo interventions, any alternative trauma-focused or non-trauma-focused psychological or bio-psychological intervention.

Outcome measures: Outcome can be measured in terms of an improvement in or reduction of PTSS. This must be measured using a previously validated measure, which could either be the primary or secondary outcome measure of a study and reported using any statistical parameter. Studies will be included if they have used one or more validated instrument/measure to

For peer review only - http://bmjopen.bmj.com/site/about/guidelines.xhtml

ascertain PTSS: e.g. Impact of Event Scale (original, revised or abbreviated version)[29-30], Clinician Administered PTSD scale (CAPS)[31], Post-Traumatic Stress Disorder Checklist for DSM-4 (PCL)[32], Post-Traumatic Stress Disorder Checklist for DSM-5 (PCL-5)[33], the Post-Traumatic Stress Diagnostic Scale (PDS)[34], or the Acute Stress Disorder Scale[35], or any other validated measures to ascertain PTSS.

Study type: Any type of controlled intervention study will be eligible. Both randomised controlled trials (RCT) and non-randomised controlled studies, evaluating the effect of non-surgical and non-pharmacological interventions on PTSS, will be included.

Measures of effect

Mean between-group difference and/or standardised mean difference will be extracted with accompanying 95% confidence intervals (CI) and p-values where this is reported. Group effect size will be extracted and reported. Symptoms of PTSS will be ascertained using standardised mean differences (continuous) and diagnosis of PTSS using risk ratio (dichotomous).

Exclusion criteria

1. Aged <18 years).
2. Single case studies, case reports alongside any review articles, clinical guidelines, letters, editorials, studies with only abstracts and any other literature with no full text availability and articles not published in the English language will be excluded.

Preparing for eligibility screening

Before screening against eligibility criteria commences, search results retrieved from the outlined electronic databases will be assembled into a digital library and categorised by the search database using the reference management software EndNote (version X20, Clarivate

For peer review only - http://bmjopen.bmj.com/site/about/guidelines.xhtml

For peer review only

Analytics, Philadelphia, PA). Any duplicate articles will be identified and eliminated at this stage.

Study selection

In the first reviewing round, two reviewers (FJ, DE) will independently screen title and abstract within the digital library potentially meeting the pre-determined inclusion criteria. Both reviewers will then independently select potential eligible articles for full text screening and apply eligibility criteria to select appropriate articles for inclusion in the review. Any disagreement will be resolved by consensus. If a resolution is not reached, a third reviewer (DF) will arbitrate any disagreement over study eligibility and resolve through discussion. Table 1 shows a checklist based on study eligibility criteria to ensure that studies are correctly identified and classified appropriately. A (PRISMA-P) flowchart will be generated and will document the selection process (inclusion and exclusion) along with reasons for exclusions.

Table 1: Eligibility criteria

**Study design**	▪ Randomised controlled trials<br>▪ Non-randomised controlled studies
**Study characteristics**	▪ Study identified via electronic database search, grey literature, google scholar, or reference lists of eligible studies<br>▪ Full text article available
**Participants**	▪ Adults aged (≥18 years) who have sustained a physically traumatic event resulting in at least one or more musculoskeletal injuries<br>▪ Studies with categorised aged group >90% of participants must be adults (≥18 years)
**Outcome measures**	▪ PTSS measured using one or more validated instrument/measures<br>▪ Any other validated measures to ascertain PTSS

Patient and public involvement

No patients or members of the public were directly involved in the design, writing or editing of this systematic review protocol. We will present the results of this review to our

For peer review only - http://bmjopen.bmj.com/site/about/guidelines.xhtml

established patient and public involvement group at the Centre of Precision Rehabilitation for Spinal Pain (CPR Spine), University of Birmingham, United Kingdom.

Data extraction (selection and coding)

Data will be managed using EndNote version 20 software (Clarivate Analytics, Philadelphia, PA). This will enable reviewer's ease of access, removal of duplicates and the review and storage of full texts and abstracts. Two reviewers will extract data of the included studies independently. Any disagreement over the eligibility of a study will be resolved through discussions and involve a third reviewer if needed. For missing data, up to two attempts will be made to contact study authors by email and/or phone to obtain further information. The following data items will be extracted from each study: authors and year of publication, study location, study design, participant's characteristics, and outcomes of interest (diagnosis or reduction in PTSS), sample size, follow-up time, setting, and items associated with risk of bias, summary statistics and methods for statistical analysis. Data to be extracted will include: title of study, first author, year and country of publication, study sample size, demographic characteristics of the study sample (e.g. age, gender, ethnicity, marital status, and education level), description of the intervention(s), description of the comparator/control group(s), duration of the study, diagnostic tool(s) used to assess PTSS, intervention effect estimates on PTSS, proportion of eligible patients who agreed to participate, descriptors of compliance with the intervention, and PTSS outcome measure status (primary or secondary). Two reviewers will independently conduct data extraction from each study using a pre-defined data extraction sheet. Extracted outcome data will be pre- and post-intervention mean and standard deviation (SD) or risk ratio. Data presented as medians or alternate measures of spread will be converted to mean and SD. When only figures are presented (rather than numerical data within text), data will be extracted and analysed where possible using software tool such as Web Plot Digitizer[36].

Risk of bias (quality) assessment

The Cochrane Risk of Bias tool V2 (RoB 2)[37] will be used to assess the risk of bias of each of the included randomised trials. Risk of bias may include selection bias (random sequence generation and allocation concealment), performance bias (blinding of patients/research team), detection bias (blinding of outcome assessment), attrition bias (incomplete outcome data), and reporting bias (selective outcome reporting). The Risk Of Bias in Non-randomised Studies of Interventions (ROBINS-I)[38] tool will be used to assess the risk of bias of NRSIs. Two reviewers (FJ, DE) will be involved in the quality assessment and any disagreements will be resolved through a third reviewer (DF). The Grading of Recommendations Assessment, Development, and Evaluation (GRADE)[39] approach will be used to assess the quality of the pooled evidence. Bias will be assessed as high, uncertain, or low risk. The risk of bias judgements will be taken into account in the final consideration of treatment effect, and studies with a high risk of bias will be flagged.

Strategy for data synthesis

Meta-analyses on reported outcomes will be performed depending upon the extent of between-study variation in participant populations, interventions, study design outcomes and methodological rigour and the number of studies reporting similar effect measures using the same assessment measures. A pairwise random-effects meta-analysis will be conducted depending upon the effect measures reported in the studies and similarities between individual studies, interventions and outcomes and the statistical heterogeneity, the assessment of whether genuine differences exist between results is low[40]. Heterogeneity will be evaluated using the I^2 statistical analysis. An I^2 of 50% and above is considered a high level of statistical heterogeneity[41]. Meta-analysis will be performed if heterogeneity between the studies is low (I^2<50%). Standardised mean Difference (SMD) and 95% CI will be extracted and reported as effect estimates of scales assessing PTSS. Standardised Mean Difference (SMD) and associated Cohen's D where available will be extracted and reported or calculable using

For peer review only - http://bmjopen.bmj.com/site/about/guidelines.xhtml

Cohen's D formula, effect size will be defined as small (0.0-0.2), medium (0.3-0.7) and large (>0.8). 95% CI will also be calculated where possible. Dichotomous outcome (i.e. presence of PTSS) will be pooled separately and risk ratios with accompanying 95% CI extracted or calculable from raw data in exposed and comparator groups. If the level of heterogeneity and risk of bias is high between studies and pooled analysis of the studies is not possible, a narrative summary of the outcome of the selected studies will be undertaken and presented in the final review. Publication bias and small study effects (funnel plot asymmetry) will be assessed by the regression-based tests proposed by Egger and Debray[42]. All analyses will be conducted in Stata 17.0 (Stata Corp, College Station, TX, USA).

Heterogeneity assessment

Assessment of the between-study heterogeneity will be statistically explored by univariate and multivariate meta-regression analyses, statistical significance will be set at ($p<0.05$). Differences in study characteristics (methodological diversity) and study populations (clinical diversity) will be further examined to explore sources of heterogeneity. Statistically significant predictors from the univariate analysis will be included in a multivariate meta-regression model, and the statistical significance will be determined at $p < 0.05$. Meta-regression will be performed in STATA using the 'metareg' command[43].

Sub-group analyses

Sub-group analyses will be performed depending on the number of studies identified. Sub-group analyses will be performed to consider the following; [1] type of intervention; [2] type of traumatic event. The level of heterogeneity between studies will be examined using the Cochrane Q-test and quantified by I^2statistical test with 95% CI. Depending on the level of heterogeneity and study characteristics, both fixed and random effect models will be used as summary effect estimates. The Mantel- Haenszel method will be performed for fixed effect model if tests between study heterogeneity is not found[44]. On the other hand the DerSimonian

For peer review only - http://bmjopen.bmj.com/site/about/guidelines.xhtml

and Laird method will be used for random effects modelling if between-study heterogeneity is anticipated because of variations in clinical measurements (measures assessing psychological intervention), study population, type of traumatic event or regions across studies[45]. This method incorporates a measure of the heterogeneity between studies. A minimum of two studies are generally considered sufficient to perform a meta-analysis[43].

Sensitivity analysis

We will conduct sensitivity analyses to assess the methodological quality and potential sources of heterogeneity of the included studies. Sources of variations may include assessment of psychological interventions, sampling strategy, adequate response and traumatic event. These will be stratified and separate sensitivity analyses conducted. A further analysis will be conducted excluding any studies with high risk of bias. We will explore the effect of restricting to studies with a low overall risk of bias.

Narrative synthesis

If the level of heterogeneity is high between studies and a meta-analysis is not feasible, we will carry out a narrative summary of the outcome of the selected studies and examined in more detail and presented in the final review outlining the reasons for the results reported in each study.

Publication bias and overall quality of the evidence

Presence of publication and small sample bias will be examined using the Egger test and inverted funnel plot technique[46]. The trim and fill method will be used to examine the magnitude of publication bias and estimate the overall effect of the remaining studies once studies which cause the funnel asymmetry are removed and then missing effects will be imputed until the funnel plot is symmetrical[47]. The STATA command metatrim will be used to perform the non-parametric trim and fill technique[48].

For peer review only

For peer review only - http://bmjopen.bmj.com/site/about/guidelines.xhtml

Certainty of evidence

To assess the certainty of evidence for the outcome of interest across studies, The GRADE framework will be used[39]. Consistent with the GRADE approach, the quality of the certainty of evidence will be assessed as high, low moderate and very low. According to the GRADE five domain, we will assess imprecision, inconsistency, indirectness, risk of bias including publication bias based on GRADE. We will evaluate imprecision, inconsistency, indirectness, risk of bias including publication bias. Applicability of the results based on the study population will also be rated when making judgement about the quality of evidence presented in the included studies[49]. The minimum number of studies recommended when examining publication bias is ten[50].

DISCUSSION

This review will comprehensively explore the effect of early intervention on PTSS, delivered within three months of a traumatic event that caused musculoskeletal injuries. The findings of this review will provide estimates for the effectiveness of evaluated interventions in terms of improvement in or reduction of PTSS severity. The relative strengths and limitations identified in the included studies will be presented in the review. The strengths of this review will include an in-depth search strategy structured around the main concepts being examined in this review, and adapted for each search database, and robust quality appraisal of research-based evidence and heterogeneity assessment to evaluate studies included in this review. Some potential limitations are likely to include between-study heterogeneity in terms of PTSS diagnostic methods, descriptions of traumatic event, setting and/or country of study and publication bias. Furthermore, PTSS commonly occurs with comorbidities and we will describe these in our review. A narrative summary of the study findings will be presented to overcome these issues if the results cannot be pooled.

Implications of results

For peer review only - http://bmjopen.bmj.com/site/about/guidelines.xhtml

This systematic review will provide a synthesis of the available literature exploring the effect of early psychological intervention on PTSS in adults (aged ≥18 years) who have experienced a traumatic event that caused musculoskeletal injuries. Findings of this review have the potential to assist clinicians to decide which interventions, either standalone or in combination, have the greatest effect on PTSS when delivered within three months of a traumatic event, and may provide the opportunity for tailoring future treatment for patients experiencing PTSS.

Ethics and dissemination

This review does not require ethical approval as only existing published data, available in scientific databases, will be used. Findings of this systematic review will be published in peer reviewed journals and presented at conferences. Any data generated from this systematic review will be made available from the corresponding author on reasonable request.

Author Contributions

FJ, DE and DF contributed equally to the conception of this protocol. FJ conceived the study design. FJ drafted the first and subsequent versions of the protocol which were reviewed/revised by DE and DF. The search strategy was developed by FJ and iteration discussed with DE and DF. The final version was approved by DE and DF. The search will be performed by FJ. FJ and DE will perform initial screening for study selection. FJ and DE will collect data from the included studies and conduct quality assessment. FJ and DE will perform data analysis/synthesis. DF will ensure data extraction consistency. FJ, DE and DF drafted and critically reviewed the manuscript and approved the final version. DF is guarantor.

Funding Statement

This project is funded by the National Institute for Health Research (NIHR) Surgical Reconstruction and Microbiology Research Centre (SRMRC). SRMRC is a research

For peer review only - http://bmjopen.bmj.com/site/about/guidelines.xhtml

infrastructure for trauma research jointly funded by the NIHR, and the ministry of defence. The views expressed are those of the author(s) and not necessarily those of the NIHR or the Department of Health and Social Care. Award number: N/A.

Competing Interests Statement

The authors have no competing interests.

Patient consent for publication

Not required.

Provenance and peer review

Not commissioned; externally peer reviewed.

For peer review only

For peer review only - http://bmjopen.bmj.com/site/about/guidelines.xhtml

REFERENCES

1. Rees S, Silove D, Chey T et al. Lifetime prevalence of gender-based violence in women and the relationship with mental disorders and psychosocial function. *JAMA.* 2011; 306:513–21.

2. O'Donnell ML, Creamer M, Pattison P, Atkin C. Psychiatric morbidity following injury. *Am J Psychiatry.* 2004; 161:507–14.

3. Lancaster CL, Teeter JB, Gros DF, Back SE. Post-traumatic stress disorder: Overview of evidence based assessment and treatment. *J Clin Med.* 2016; 5: 105

4. Shalev AY, Gevonden M, Ratanatharathorn A et al. Estimating the risk of PTSD in recent trauma survivors: Results of the international consortium to predict PTSD (ICPP). *World J Psychiatry*. 2019; 18:77–87.

5. Kavan MG, Elsasser GN, Barone E. The Physician role in managing acute stress disorder. *Am Fam Physician*. 2012; 86:643-649.

6. American Psychiatric Association. (2013). Diagnostic and Statistical Manual of Mental Disorders. (5th ed). American Psychiatric Association; Arlington, VA, USA. Retrieved from: https://psychiatry.org/psychiatrists/practice/dsm.

7. Nyvold O, Nygaard E, Augusti EM et al. Tamnes, C. K. Unity or diversity of executive functioning in children and adolescents with post-traumatic stress symptoms? A systematic review and meta-analysis. Child Neuropsychol. 2022; 28:374-393.

8. Ruiz-Parraga GT, Lopez-Martinez AE. The contribution of postraumatic stress symptoms to chronic pain adjustments. Health Psychol. 2014; 33:958-67.

9. Lee C, Porter KM. Prehospital management of lower limb fractures. Emerg Med J. 2005; 22:660–3

10. Accidents and injuries statistics: Causes of death - deaths by country of residence and occurrence. Eurostat; 2017. Available: https://ec.europa.eu/eurostat/databrowser/view/hlth_cd_aro/default/table?lang=en

11. Global status report on road safety 2018. Geneva: World Health Organization; 2018. Available: https://www.who.int/violence_injury_prevention/road_safety_status/2018/en/

12. National Audit Office Major Trauma Care in England 2010. London: The Stationery Office. Available: https://www.nao.org.uk/report/major-trauma-care-in-england/

13. Davenport RA, Tai N, West A, et al. A major trauma centre is a specialty hospital not a hospital of specialties/ *Br J Surg*. 2010; 97:109–117.

For peer review only

For peer review only - http://bmjopen.bmj.com/site/about/guidelines.xhtml

14. Herron J, Hutchinson R, Lecky F, Bouamra O, Edwards A, Woodford M, et al. The impact of age on major orthopaedic trauma: an analysis of the United Kingdom trauma audit research network database. *Bone Joint J.* 2017; 99-B (12):1677–80.

15. Trauma and Audit Research Network. Modelling Trauma Workload: A Project for the Department of Health, 2009. Available: https://www.nao.org.uk/wp-content/uploads/2010/02/0910213.pdf

16. Sparks SW. Posttraumatic Stress Syndrome: What Is It?, J Trauma Nurs. 2018; 25:60-65

17. American Psychiatric Association. Diagnostic and Statistical Manual of Mental Disorders, (5th ed). Arlington, VA: American Psychiatric Publishing (2013).

18. Alhussaini NW, Riaz M. Effectiveness of pharmacological and psychological interventions for treating post-traumatic stress disorder in adults with childhood abuse: protocol for a systematic review and network meta-analysis. *BMJOpen.* 2021; 11:e048790.

19. Jonas DE, Cusack K, Forneris CA et al. Psychological and pharmacological treatments for adults with posttraumatic stress disorder (PTSD). AHRQ comparative effectiveness reviews. Rockville (MD: Agency for Healthcare Research and Quality, 2013. www.effectivehealthcare.ahrq.gov/reports/final.cfm.

20. Bisson JI, Shepherd JP, Joy D. Early cognitive-behavioural therapy for post-traumatic stress symptoms after physical injury. Randomised controlled trial. *Br J Psychiatry*. 2004;184:63-9

21. Kitchiner NJ, Lewis C, Roberts NP. Active duty and ex-serving military personnel with post-traumatic stress disorder treated with psychological therapies: systematic review and meta-analysis. *Eur J Psychotraumatol*. 2019; 8: 1684226

22. Wu KK, Li FW, Cho VW. A randomized controlled trial of the effectiveness of brief-CBT for patients with symptoms of posttraumatic stress following a motor vehicle crash. *Behav Cogn Psychother*. 2014; 42:31-47.

23. Shamseer L, Moher D, Clarke M. PRISMA-P Group. Preferred reporting for systematic review and meta-analysis protocols (PRISMA-P). *BMJ* 2015; 349: g7647.

24. McKenzie JE & Brennan SE. Chapter 12: Synthesizing and presenting findings using other methods. In: Higgins JPT, Thomas J, Chandler J, Cumpston M, Li T, Page MJ, Welch VA (editors). Cochrane Handbook for Systematic Reviews of Interventions version 6.2 (updated February 2021). Cochrane, 2021.

For peer review only - http://bmjopen.bmj.com/site/about/guidelines.xhtml

25. Busch FN, Milrod BL: Trauma-focused psychodynamic psychotherapy. *Psychiatr Clin North Am*. 2018; 41:277–287

26. Donker T, Griffiths KM, Cuijpers P et al. Psychoeducation for depression, anxiety and psychological distress: a meta-analysis. *BMC Med*. 2009; 16:7-79

27. Moloud R, Saeed Y, Mahmonir H et al. Cognitive-behavioral group therapy in major depressive disorder with focus on self-esteem and optimism: an interventional study. *BMC Psychiatry*. 2022; 28:299

28. Shapiro, F. The Role of Eye Movement Desensitization and Reprocessing (EMDR) Therapy in Medicine: Addressing the Psychological and Physical Symptoms Stemming from Adverse Life Experiences. *Pem J*. 2014; 18:71-77.

29. Roland M, Morris R. A study of the natural-history of low-back-pain 2. Development of guidelines for trials of treatment in primary care. *Spine* 1983; 8:145–50.

30. Thoresen S, Tambs K, Hussain A, Heir T, Johansen VA, Bisson JI. Brief measure of posttraumatic stress reactions: impact of Event Scale-6. *Soc Psychiatry Psychiatr Epidemiol* 2010; 45:405-12.

31. Blake DD, Weathers F, Nagy L, et al. The development of a clinician-administered PTSD scale. *J Trauma Stress* 1995; 8:75–90.

32. Blanchard EB, Jones-Alexander J, Buckley TC et al. Psychometric properties of the PTSD Checklist (PCL). Behav Res Ther 1996; 34: 669-673

33. Weathers FW, Litz BT, Keane TM, et al. (2014, February 5). PTSD Checklist for DSM-5 (PCL-5). National Center for PTSD; 2014. Available: http://www.ptsd.va.gov/professional/assessment/adult-sr/ptsd-checklist.asp

34. Foa EB, McLean CP, Zang Y et al. Psychometric properties of the Posttraumatic Diagnostic Scale for DSM-5 (PDS-5). *Psychol Assess* 2016; 28: 1166-1171.

35. Bryant RA, Moulds ML, Guthrie RM. Acute stress disorder scale: a self-report measure of acute stress disorder. *Psychol Assess* 2000; 12:61-8

36. Rohatgi A. WebPlotDigitizer - Extract data from plots, images, and maps. 2020. Available: https://automeris.io/WebPlotDigitizer/

37. Sterne AC, Savović J, Page MJ et al. RoB 2: a revised tool for assessing risk of bias in randomised trials. *BMJ.* 2019; 366: l4898.

38. JAC Sterne, MA Hernán, BC Reeves et al. ROBINS-I: a tool for assessing risk of bias in non-randomised studies of interventions. BMJ. 2016; 355:i4919.

For peer review only - http://bmjopen.bmj.com/site/about/guidelines.xhtml

39. Balshem H, Helfand M, Schünemann H.J et al. GRADE guidelines: 3. Rating the quality of evidence. J. Clin. Epidemiol 2011; 64:401-40.

40. Higgins JPT, Thomas J, Chandler J, Cumpston M, Li T, Page MJ, Welch VA (editors). Cochrane Handbook for Systematic Reviews of Interventions version 6.3 (updated February 2022). Cochrane, 2022. Available: www.training.cochrane.org/handbook.

41. Higgins JP, Green S. Cochrane handbook for systematic reviews of interventions, 2011. Available: http://www.mri.gov.lk/assets/Uploads/Research/Cochrane-Hand-booktext.pdf.

42. Egger M, Davey Smith G, Schneider M, Minder C. Bias in meta-analysis detected by a simple, graphical test. BMJ. 1997;315(7109):629–34.

43. Harbord RM, Higgins JPT. Meta-regression in Stata. Stata Journal 2008; 8:493-419. Available: http://www.stata-journal.com/article.html?article=sbe23_1

44. Mantel N, Haenszel W. Statistical aspects of the analysis of data from retrospective studies of disease. *J Natl Cancer Inst* 1959; 22:719-48.

45. DerSimonian R, Laird N. Meta-analysis in clinical trials. *Control Clin Trials* 1986; 7: 177-188.

46. Egger M , Smith GD , Schneider M , et al . Bias in meta-analysis detected by a simple, graphical test. *BMJ* 1997;315:629–34.

47. Duval S, Tweedie RL. Trim and fill: A simple funnel plot-based method of testing and adjusting for publication bias in meta-analysis. *Biometrics* 2000b; 56:276-284.

48. Sterne JAC, Newton HJ, Cox NJ. Meta-Analysis in Stata: An Updated Collection from the Stata Journal. 2009; College Station, TX, Stata Press.

49. Guyatt G, Oxman AD, Akl EA, et al. GRADE guidelines: 1. Introduction-GRADE evidence profiles and summary of findings tables. *J Clin Epidemiol* 2011; 64:383–94.

50. Sterne JA, Sutton AJ, Ioannidis JPA. Recommendations for examining and interpreting funnel plot asymmetry in meta-analyses of randomised controlled trials. *BMJ* 2011; 343: d4002.

For peer review only - http://bmjopen.bmj.com/site/about/guidelines.xhtml

Supplementary file 1: PRISMA-P Checklist: This checklist has been adapted for use with protocol submissions to *Systematic Reviews* from Table 3 in Moher D et al: Preferred reporting items for systematic review and meta-analysis protocols (PRISMA-P) 2015 statement. *Systematic Reviews* 2015 **4**:1

Early interventions for post-traumatic stress following musculoskeletal trauma: Protocol for a systematic review and meta-analysis

Section/topic	#	Checklist item	Information reported		Page number(s)
			Yes	No	
**ADMINISTRATIVE INFORMATION**					
**Title**					
Identification	1a	Identify the report as a protocol of a systematic review	☒	☐	1
Update	1b	If the protocol is for an update of a previous systematic review, identify as such	☐	☒	N/A
**Registration**	2	If registered, provide the name of the registry (e.g., PROSPERO) and registration number in the Abstract	☒	☐	2
**Authors**					
Contact	3a	Provide name, institutional affiliation, and e-mail address of all protocol authors; provide physical mailing address of corresponding author	☒	☐	1
Contributions	3b	Describe contributions of protocol authors and identify the guarantor of the review	☒	☐	14
**Amendments**	4	If the protocol represents an amendment of a previously completed or published protocol, identify as such and list changes; otherwise, state plan for documenting important protocol amendments	☐	☒	N/A
**Support**					
Sources	5a	Indicate sources of financial or other support for the review	☒	☐	14
Sponsor	5b	Provide name for the review funder and/or sponsor	☒	☐	14
Role of sponsor/funder	5c	Describe roles of funder(s), sponsor(s), and/or institution(s), if any, in developing the protocol	☒	☐	14
**INTRODUCTION**					
**Rationale**	6	Describe the rationale for the review in the context of what is already known	☒	☐	4-5
**Objectives**	7	Provide an explicit statement of the question(s) the review will address with reference to participants, interventions, comparators, and outcomes (PICO)	☒	☐	6-7
**METHODS**					
**Eligibility criteria**	8	Specify the study characteristics (e.g., PICO, study design, setting, time frame) and report characteristics (e.g., years considered, language, publication status) to be used as criteria for eligibility for the review	☒	☐	6-8
**Information sources**	9	Describe all intended information sources (e.g., electronic databases, contact with study authors, trial	☒	☐	6

BioMed Central The Open Access Publisher

For peer review only

Section/topic	#	Checklist item	Information reported Yes	No	Page number(s)
		registers, or other grey literature sources) with planned dates of coverage			
**Search strategy**	10	Present draft of search strategy to be used for at least one electronic database, including planned limits, such that it could be repeated	☒	☐	6
STUDY RECORDS					
Data management	11a	Describe the mechanism(s) that will be used to manage records and data throughout the review	☒	☐	8
Selection process	11b	State the process that will be used for selecting studies (e.g., two independent reviewers) through each phase of the review (i.e., screening, eligibility, and inclusion in meta-analysis)	☒	☐	8
Data collection process	11c	Describe planned method of extracting data from reports (e.g., piloting forms, done independently, in duplicate), any processes for obtaining and confirming data from investigators	☒	☐	9-10
**Data items**	12	List and define all variables for which data will be sought (e.g., PICO items, funding sources), any pre-planned data assumptions and simplifications	☒	☐	6-7
**Outcomes and prioritization**	13	List and define all outcomes for which data will be sought, including prioritization of main and additional outcomes, with rationale	☒	☐	7
**Risk of bias in individual studies**	14	Describe anticipated methods for assessing risk of bias of individual studies, including whether this will be done at the outcome or study level, or both; state how this information will be used in data synthesis	☒	☐	10
DATA					
**Synthesis**	15a	Describe criteria under which study data will be quantitatively synthesized	☒	☐	10-11
	15b	If data are appropriate for quantitative synthesis, describe planned summary measures, methods of handling data, and methods of combining data from studies, including any planned exploration of consistency (e.g., I^2, Kendall's tau)	☒	☐	10-11
	15c	Describe any proposed additional analyses (e.g., sensitivity or subgroup analyses, meta-regression)	☒	☐	11-12
	15d	If quantitative synthesis is not appropriate, describe the type of summary planned	☒	☐	11-12
**Meta-bias(es)**	16	Specify any planned assessment of meta-bias(es) (e.g., publication bias across studies, selective reporting within studies)	☒	☐	12-13
**Confidence in cumulative evidence**	17	Describe how the strength of the body of evidence will be assessed (e.g., GRADE)	☒	☐	13

For peer review only

BioMed Central The Open Access Publisher

Early interventions for post-traumatic stress following musculoskeletal trauma: Protocol for a systematic review and meta-analysis

Medline search strategy

1. Post-traumatic stress symptom

Post-traumatic stress.mp **OR** Post-traumatic stress disorder.mp **OR** Post traumatic.mp **OR** Post-traumatic stress symptom.mp **OR** Stress symptoms.mp **OR** Post-trauma*.mp **OR** PTSS.mp or PTSD.mp **OR** Acute stress disorder.mp **OR** ASD.mp

2. Musculoskeletal trauma

Musculoskeletal trauma.mp **OR** musculoskeletal injury.mp **OR** Orthopaedic trauma.mp **OR** Orthopaedic injuries.mp **OR** Orthopedic trauma.mp **OR** Orthopedic injury.mp **OR** Musculoskeletal.mp **OR** Musculoskeletal inj* **OR** Lower extremity inj* **OR** Upper extremity inj* **OR** Exp fractures, bone **OR** Exp whiplash injuries **OR** Whiplash.mp **OR** Athletic injuries.mp **OR** Traumatic injuries.mp **OR** Falls.mp **OR** Traffic collision.mp **OR** Exp multiple trauma **OR** Traumatic inj* **OR** Gunshot injury* **OR** Stab wound*

3. Intervention

Psychotherapies.mp **OR** psychotherap* **OR** physiotherap* **OR** physical therap* **OR** emdr.mp **OR** eye movement desensiti* **OR** prevention.mp **OR** intervention.mp **OR** therap* **OR** psychoeducation.mp **OR** treatment.mp **OR** cognitive behavioural therapy.mp **OR** CBT.mp **OR** rehabilitation.mp **OR** imagery.mp

For peer review only - http://bmjopen.bmj.com/site/about/guidelines.xhtml

CINAHL search strategy

1. Post-traumatic stress symptom

Post traumatic **OR** Post-traumatic stress symptom **OR** Post-trauma **OR** PTSS **OR** Post-traumatic stress **OR** Post-traumatic stress disorder **OR** Post-traumatic stress symptom **OR** PTSD **OR** Acute stress disorder **OR** ASD

2. Musculoskeletal trauma

Musculoskeletal trauma **OR** Musculoskeletal injury **OR** Orthopaedic trauma **OR** Orthopaedic injury **OR** Orthopedic trauma **OR** Lower extremity injury **OR** Upper extremity injury **OR** Fractures, bone **OR** Whiplash injuries **OR** Whiplash **OR** Athletic injury **OR** Multiple trauma **OR** Traumatic injury **OR** Traumatic injuries **OR** Falls **OR** Gunshot injury **OR** Stab wound **OR** Traffic collision

3. Intervention

Psychotherapies **OR** psychotherapy **OR** physiotherapy **OR** physical therapy **OR** EMDR **OR** eye movement desensitisation **OR** prevention **OR** intervention **OR** therapy **OR** psychoeducation **OR** treatment **OR** cognitive behavioural therapy **OR** CBT **OR** rehabilitation **OR** imagery

For peer review only - http://bmjopen.bmj.com/site/about/guidelines.xhtml

EMBASE search strategy

1. Post-traumatic stress symptom

Post traumatic.ab, ti **OR** Post-traumatic stress symptom$.ab, ti **OR** Post-trauma.mp **OR** PTSS.mp **OR** Post traumatic stress.mp **OR** Post-traumatic stress disorder.mp **OR** Post traumatic.mp **OR** Post-traumatic stress symptom.mp **OR** PTSD.mp **OR** Acute stress disorder.mp **OR** ASD.mp

PTSS.tw

2. Musculoskeletal trauma

Musculoskeletal trauma.mp **OR** musculoskeletal injury.mp **OR** Orthopaedic trauma.mp **OR** orthopedic trauma.mp **OR** Fractures, bone.mp **OR** Whiplash injuries.mp **OR** Whiplash.mp **OR** Multiple trauma.mp **OR** Traumatic injuries.mp **OR** Falls.mp **OR** Gunshot injury.mp **OR** Stab wound.mp **OR** Traffic collision.mp **OR** Musculoskeletal inj$.ab,ti **OR** Orthopaedic inj$.ab,ti **OR** Orthopedic inj$.ab,ti **OR** Lower extremity inj$.ab,ti **OR** Upper extremity inj$.ab,ti **OR** Athletic inj$.ab,ti **OR** Traumatic inj$.ab,ti

3. Intervention

Psychotherap$.ab.ti **OR** Psychotherapies.mp **OR** Psychotherapy.mp **OR** physiotherapy$.ab.ti **OR** Physiotherapies.mp **OR** Physiotherapy.mp **OR** physical therap$.ti.ab **OR** physical therapies.mp **OR** physical therapy.mp **OR** EMDR.mp **OR** eye movement desensitisation.mp **OR** desensitization.mp **OR** prevention.mp **OR** intervention.mp **OR** therap$.ab.ti **OR** therapies.mp **OR** therapy.mp **OR** psychoeducation.mp **OR** treatment.mp **OR** cognitive behavioural therapy.mp **OR** CBT.mp **OR** rehabilitation.mp **OR** imagery.mp

For peer review only - http://bmjopen.bmj.com/site/about/guidelines.xhtml

PsycINFO search strategy

1. Post-traumatic stress symptom

Post-traumatic stress disorder.mp **OR** post-traumatic stress disorders.mp **OR** posttraumatic stress disorder.mp **OR** posttraumatic stress disorders.mp **OR** disorder* **OR** post-traumatic.mp **OR** post-traumatic stress symptom*.mp **OR** post traumatic stress symptom*.mp **OR** PTSS.mp **OR** PTSD.mp **OR** acute stress disorder.mp **OR** ASD.mp

2. Musculoskeletal trauma

Musculoskeletal trauma.mp **OR** musculoskeletal injur*.mp **OR** orthopaedic trauma.mp **OR** orthopaedic injur*.mp **OR** orthopedic trauma.mp **OR** lower extremity injur*.mp **OR** upper extremity injur*.mp **OR** fracture*.mp **OR** bone.mp **OR** whiplash injur*.mp **OR** whiplash **OR** athletic injur*.mp **OR** multiple trauma.mp **OR** traumatic injur*.mp **OR** traumatic injur*.mp **OR** falls.mp **OR** gunshot injur*.mp **OR** stab wound*.mp **OR** traffic collision*.mp

3. Intervention

Psychotherap* **OR** Psychotherapies.mp **OR** Psychotherapy.mp **OR** physiotherapy* **OR** Physiotherapies.mp **OR** Physiotherapy.mp **OR** physical therap* **OR** physical therapies.mp **OR** physical therapy.mp **OR** EMDR.mp **OR** eye movement desensitisation.mp **OR** desensitization.mp **OR** prevention.mp **OR** intervention.mp **OR** therap* **OR** therapies.mp **OR** therapy.mp **OR** psychoeducation.mp **OR** treatment.mp **OR** cognitive behavioural therapy.mp **OR** CBT.mp **OR** rehabilitation.mp **OR** imagery.mp

For peer review only - http://bmjopen.bmj.com/site/about/guidelines.xhtml

PubMed search strategy

1. Post-traumatic stress symptom

Post-traumatic stress **OR** Post-traumatic stress disorder **OR** Post traumatic **OR** Post-traumatic stress symptom **OR** Stress symptoms **OR** Post-trauma **OR** PTSS **OR** PTSD **OR** Acute stress disorder **OR** ASD

2. Musculoskeletal trauma

Musculoskeletal trauma **OR** musculoskeletal injury **OR** Orthopaedic trauma **OR** Orthopaedic injuries **OR** Orthopedic trauma **OR** Orthopedic injury **OR** Musculoskeletal **OR** Orthopaedic **OR** Musculoskeletal injury **OR** Lower extremity injury **OR** Upper extremity injury **OR** fractures **OR** bone **OR** whiplash injuries **OR** Whiplash **OR** Athletic injuries **OR** Traumatic injuries **OR** Falls **OR** Traffic collision **OR** multiple trauma **OR** Traumatic injury **OR** Gunshot injury OR Stab wound

3. Intervention

Psychotherapy **OR** Psychotherapies **OR** Physiotherapy **OR** Physiotherapies **OR** physical therapy **OR** physical therapies **OR** EMDR **OR** eye movement desensitisation **OR** desensitization **OR** prevention **OR** intervention **OR** therapy **OR** therapies **OR** psychoeducation **OR** treatment **OR** cognitive behavioural therapy **OR** CBT **OR** rehabilitation **OR** imagery

For peer review only - http://bmjopen.bmj.com/site/about/guidelines.xhtml

Web of Science search strategy

1. **Part 1: Post-traumatic stress symptom**

Post-traumatic stress **OR** Post-traumatic stress disorder **OR** Post traumatic **OR** Post-traumatic stress symptom **OR** Stress symptoms **OR** Post-trauma **OR** PTSS **OR** PTSD **OR** Acute stress disorder **OR** ASD

2. **Musculoskeletal trauma**

Musculoskeletal trauma **OR** musculoskeletal injury **OR** Orthopaedic trauma **OR** Orthopaedic injuries **OR** Orthopedic trauma **OR** Orthopedic injury **OR** Musculoskeletal **OR** Orthopaedic **OR** Musculoskeletal injury **OR** Lower extremity injury **OR** Upper extremity injury **OR** fractures **OR** bone **OR** whiplash injuries **OR** Whiplash **OR** Athletic injuries **OR** Traumatic injuries **OR** Falls **OR** Traffic collision **OR** multiple trauma **OR** Traumatic injury **OR** Gunshot injury OR Stab wound

3. **Intervention**

Psychotherapy **OR** Psychotherapies **OR** Physiotherapy **OR** Physiotherapies **OR** physical therapy **OR** physical therapies **OR** EMDR **OR** eye movement desensitisation **OR** desensitization **OR** prevention **OR** intervention **OR** therapy **OR** therapies **OR** psychoeducation **OR** treatment **OR** cognitive behavioural therapy **OR** CBT **OR** rehabilitation **OR** imagery

For peer review only - http://bmjopen.bmj.com/site/about/guidelines.xhtml

BMJ Open

Early interventions for post-traumatic stress following musculoskeletal trauma: Protocol for a systematic review and meta-analysis

Journal:	*BMJ Open*
Manuscript ID	bmjopen-2022-065590.R1
Article Type:	Protocol
Date Submitted by the Author:	03-Aug-2022
Complete List of Authors:	Jadhakhan, Ferozkhan; University of Birmingham, School of Sport, Exercise and Rehabilitation Sciences<br>Evans, David; University of Birmingham, School of Sport, Exercise and Rehabilitation Sciences<br>Falla, Deborah; University of Birmingham
**Primary Subject Heading**:	Rehabilitation medicine
Secondary Subject Heading:	Mental health
Keywords:	Musculoskeletal disorders < ORTHOPAEDIC & TRAUMA SURGERY, TRAUMA MANAGEMENT, Rehabilitation medicine < INTERNAL MEDICINE, Trauma management < ORTHOPAEDIC & TRAUMA SURGERY

SCHOLARONE™
Manuscripts

Early interventions for post-traumatic stress following musculoskeletal trauma: Protocol for a systematic review and meta-analysis

Ferozkhan Jadhakhan[1], David W Evans[1], Deborah Falla[1*]

1. Centre of Precision Rehabilitation for Spinal Pain, School of Sport, Exercise and Rehabilitation Sciences, College of Life and Environmental Sciences, University of Birmingham, Edgbaston, Birmingham, B15 2TT, United Kingdom

For peer review only

Correspondence:

Professor Deborah Falla
Centre of Precision Rehabilitation for Spinal Pain
School of Sport, Exercise and Rehabilitation Sciences,
College of Life and Environmental Sciences,
University of Birmingham,
Birmingham, B15 2TT, UK.
Tel: +44 121 415 4220
Email: d.falla@bham.ac.uk

ABSTRACT

Introduction: Post-traumatic stress symptoms (PTSS) can be triggered following exposure to a traumatic event, such as violence, disasters, serious accidents, and injury. Little is known about which interventions provide the greatest benefit for PTSS. This systematic review aims to estimate the effects of early interventions on PTSS following musculoskeletal trauma.

Methods/Analysis: Development of this review protocol was guided by the Preferred Reporting Items for Systematic Reviews and Meta-Analysis-P. This review will include randomised controlled trials (RCT) and non-randomised controlled studies evaluating the effect of early (within 3 months of a traumatic event) non-pharmacological and non-surgical interventions on PTSS in adults (aged ≥18 years). MEDLINE, PsycINFO, EMBASE, CINAHL, ZETOC, PROSPERO, Web of Science, PubMed and Google Scholar, as well as key journals/grey literature, will be searched from inception to 31st July 2022. Only articles published in English will be considered. Two independent reviewers will search, screen studies, extract data, and assess risk of bias using the Cochrane Risk of Bias tool V2 (RoB 2) and the Risk Of Bias in Non-randomized Studies of Interventions (ROBINS-I) respectively. Mean difference or standardised mean difference will be extracted with accompanying 95% confidence intervals (CI) and p-values where these are reported. Group effect size will be extracted and reported. Symptoms of PTSS will be ascertained using standardised mean differences (continuous) and diagnosis of PTSS using risk ratio (dichotomous). If possible, study results will be pooled into a meta-analysis. A narrative synthesis of the results will be presented if heterogeneity is high. The overall quality of evidence and risk of bias will be assessed using the GRADE, RoB 2 and ROBINS-I guidelines respectively.

Ethics and dissemination: Ethical approval is not required for this systematic review since data from published studies will be used. This review is expected to provide a better understanding of the effect of early intervention for PTSS following musculoskeletal trauma. Findings of this review will be disseminated in peer-reviewed publications and through national and international conferences.

For peer review only

For peer review only - http://bmjopen.bmj.com/site/about/guidelines.xhtml

Prospero registration: CRD42022333905

Keywords: Musculoskeletal trauma, psychological intervention, post-traumatic stress symptoms, early intervention

For peer review only

For peer review only - http://bmjopen.bmj.com/site/about/guidelines.xhtml

STRENGTHS AND LIMITATIONS

- This systematic review protocol follows the reporting guidelines according to the Preferred Reporting Items for Systematic Reviews and Meta-Analysis Protocols (PRISMA-P checklist).
- This systematic review addresses a gap in the current evidence base by providing a comprehensive assessment of the effect of early psychological interventions for PTSS following musculoskeletal trauma.
- A comprehensive search strategy with a wide spectrum of search terms will be used.
- A meta-analysis of the results may not be possible due to the high risk of bias among studies and methodological heterogeneity between them, thus a narrative summary of the outcome of the selected studies may be presented in the final review.
- Findings of this study may be limited by publication bias, study heterogeneity, the measurements used to assess PTSS, different psychological interventions (e.g. psychoeducation, cognitive behavioural therapy, and eye movement desensitisation and reprocessing) and the methodological quality of included studies.

For peer review only - http://bmjopen.bmj.com/site/about/guidelines.xhtml

INTRODUCTION

Post-traumatic stress symptoms (PTSS) can be triggered following exposure to a traumatic event, such as violence, disasters, serious accidents, and injury[1-2]. PTSS is characterised by clusters of symptoms: re-experiencing the trauma (intrusion); avoidance of stimuli related to the event; emotional numbness (dissociation); and (hyper) arousal[3]. PTSS can have a significant impact on the day-to-day life of those exposed to a traumatic event and their families,[4] affecting their social, occupational, interpersonal relationships, and physical health. If these symptoms appear within one month of a traumatic event, and certain criteria are met, acute stress disorder (ASD) may be diagnosed[5]. In most cases, PTSS resolves on its own, but for some these symptoms may persist[6]. If these symptoms persist beyond one month of the traumatic event, post-traumatic stress disorder (PTSD) may be diagnosed,[7] although this progression is not a certainty.

Musculoskeletal injuries are common and can lead to severe long-term pain and disability. They are the leading cause of severe morbidity and mortality worldwide[8]. In 2016, an estimated 146,000 deaths were attributed to road traffic accidents across the 27 countries of the European Union (EU), equivalent to 3.2% of all deaths registered for that year[9]. Globally, road traffic accidents injured more than 50 million people and killed approximately 1.35 million people in 2016; an estimated 3,700 deaths per day[10]. In England alone, an estimated 20,000 cases of major trauma occur each year resulting in 5,400 deaths with many sustaining permanent disability requiring long term care[11]. Annually in the United Kingdom (UK), between 40,000-90,000 individuals are involved in a major traumatic accident, of which 50% will have sustained a musculoskeletal injury[12-13]. Some of the most common major trauma include injuries to the head, neck, spine, chest, limbs, abdomen and pelvis. In England, the most common mechanisms of injury are road traffic accidents and falls[14]. It is estimated that the annual loss in economic output as a result of major trauma is between £3.3 and £3.7 billion in

For peer review only - http://bmjopen.bmj.com/site/about/guidelines.xhtml

the UK[11]. The annual emergency and hospital treatment for trauma care is estimated to cost the National Health Service (NHS) an estimated £300-£400 million[15].

Although ASD and PTSD are established as serious health concerns following a major physical trauma, sub-threshold PTSS are also considered a serious health issue associated with significant distress and impairment[16-17]. Most people experience some PTSS immediately following a traumatic event[18-19]. For some, symptoms develop six months or more after the initial traumatic event and rise above the PTSD threshold over time[20]. PTSS impacts both quality of life and healthcare resources, which is why there is a need to provide effective and timely intervention strategies.

Several non-pharmacological interventions have been shown to be effective in the management of PTSS. Several psychological treatment approaches have been studied, such as behavioural (exposure) therapy, trauma-focused cognitive behavioural therapy (CBT), supportive (psychodynamic and interpersonal) psychotherapy, brief eclectic psychotherapy, and other therapies performed individually or in groups[21]. In addition, some exercise-based interventions have been developed and tested, including, aerobic exercise[22], eye movement desensitisation and reprocessing (EMDR)[23], and prolonged exposure (PE)[24]. There have also been studies evaluating physical stimulation-based treatments such as massage[25] and acupuncture[26].

Most psychological treatments aim to reduce PTSS by working through stressors related to the traumatic event[27]. For example, Bisson and colleagues[28] compared four 1-hour CBT sessions to no intervention (standard care only, involving no formal intervention) in individuals with evidence of PTSS. The results showed that Impact of Event Scale (IES) scores were reduced in the intervention group at 13 months, compared to the control group (adjusted mean difference=8.4, 95% CI 2.4–14.36). On the other hand, a systematic review and meta-analysis of multiple psychological interventions designed to treat PTSD symptoms in military personnel

For peer review only - http://bmjopen.bmj.com/site/about/guidelines.xhtml

within three months of a traumatic event, found little evidence to support any interventions, although trauma-focused CBT was associated with a reduction in PTSD symptoms post-treatment compared to waitlist-control (standardised mean difference (SMD) −1.22; 95% CI, −1.78 to −0.66)[29]. Exercise-based interventions are purported to act upon PTSS by reducing stress through the activation of endorphins[30] and potentially increasing self-efficacy[31]. Stimulation-based interventions also plausibly work through general stress reduction[32-35]. Results from a meta-analysis of four RCTs, examining the effect of exercise (resistance training and walking) on PTSD symptoms compared to a control group, revealed that physical activity significantly reduced symptoms of PTSD, compared to control groups (Hedges' g = -0.35, 95% CI [-0.63 to -0.07], p=0.02)[36].

Many of these studies have potential methodological shortcomings, justifying a robust review of empirical evidence evaluating non-pharmacological interventions in individuals with PTSS following musculoskeletal trauma. Furthermore, because of the wide range of available non-pharmacological interventions used to improve PTSS, it is important to identify which intervention(s), if any, whether alone or in combination have the greatest effect on PTSS when applied early after (within three months of) the traumatic event. To date, no systematic review has investigated the effect of early non-pharmacological interventions for PTSS following musculoskeletal trauma.

Aims

This systematic review aims to:

- Synthesise evidence evaluating the effect of early non-pharmacological, non-surgical interventions on PTSS, when commenced within 3 months after a traumatic event causing musculoskeletal injuries
- Establish which interventions are more effective at reducing PTSS severity
- Outline implications for clinical practice

For peer review only - http://bmjopen.bmj.com/site/about/guidelines.xhtml

METHODS

This systematic review protocol follows the reporting guidelines according to the Preferred Reporting Items for Systematic Reviews and Meta-Analysis Protocols (PRISMA-P checklist)[37] (Supplementary File 1). This review protocol was registered with the International Prospective Register of Systematic Reviews (PROSPERO registration: CRD42022333905) on the 19th of May 2022 (https://www.crd.york.ac.uk/prospero/export_details_pdf.php). The planned start date for this review is 31st July 2022 and the planned end date is 30th September 2022.

Search strategy

The following citation databases MEDLINE, PsycINFO, EMBASE, CINAHL, ZETOC, PROSPERO, Web of Science, PubMed, Google Scholar and Cochrane central register of controlled trials as well as key journals/grey literature will be searched from inception to 31st July 2022. Due to available resources and feasibility (availability of translators within the department), only articles published in English will be considered eligible. The following key words will be used to retrieve relevant articles: trauma, musculoskeletal, acute stress disorder, post-traumatic stress, ASD, PTSD, PTSS, therapy, intervention, cognitive behavioural, EMDR, psychological, exercise, manual therapy, massage, acupuncture. A search strategy for each database can be found in Supplementary File 2.

Inclusion criteria

The selection criteria for inclusion/exclusion of studies will follow the Participants, Interventions, Comparators, Outcomes and Study design (PICOS) framework[38].

Population: Individuals aged ≥18 years who sustained a physically traumatic event that resulted in one or more musculoskeletal injuries. In studies with categorised aged groups, >90% of participants must be adults (≥18 years).

For peer review only - http://bmjopen.bmj.com/site/about/guidelines.xhtml

Intervention: At least one non-pharmaceutical and non-surgical intervention must be applied within 3 months of the traumatic event. Types of intervention may include, for example: trauma-focused psychodynamic psychotherapy[39], psychoeducation[40], cognitive behavioural therapy (CBT)[41], eye movement desensitisation and reprocessing (EMDR)[42], exercise[43], manual therapy[44], and acupuncture[45]. This review will also include any trials where a combination of two or more eligible interventions (e.g. CBT and aerobic exercise) were evaluated.

Comparator(s)/control: Comparator group(s) can be: true control (i.e. no intervention provided), wait-list controls, care as usual, placebo interventions, pharmaceutical or surgical interventions, any alternative trauma-focused or non-trauma-focused psychological or physical intervention.

Outcome measures: Outcome can be measured in terms of an improvement in or reduction of PTSS. This must be measured using a previously validated measure, which could either be the primary or secondary outcome measure of a study and reported using any statistical parameter. Studies will be included if they have used one or more validated instrument/measure to ascertain PTSS: e.g. Impact of Event Scale (original, revised or abbreviated version)[46-47], Clinician Administered PTSD scale (CAPS)[48], Post-Traumatic Stress Disorder Checklist for DSM-4 (PCL, any version)[49-50], Post-Traumatic Stress Disorder Checklist for DSM-5 (PCL-5)[51], the Post-Traumatic Stress Diagnostic Scale (PDS)[52], or the Acute Stress Disorder Scale[53], or any other validated measure of PTSS.

Study type: Any type of controlled intervention study will be eligible. Both randomised controlled trials (RCT) and non-randomised controlled studies, evaluating the effect of non-pharmacological and non-surgical interventions on PTSS, will be included.

For peer review only - http://bmjopen.bmj.com/site/about/guidelines.xhtml

Exclusion criteria

1. Aged <18 years
2. Single case studies, case reports alongside any review articles, clinical guidelines, letters, editorials, studies with only abstracts and any other literature with no full text availability and articles not published in the English language will be excluded.
3. Studies focusing solely on patients with traumatic brain injury (TBI), spinal cord injuries, burns or deliberately self-injured patients

Measures of effect

Mean between-group difference and/or standardised mean difference will be extracted with accompanying 95% confidence intervals (CI) and *p*-values where this is reported. Group effect size will be extracted and reported. Symptoms of PTSS will be ascertained using standardised mean differences (continuous) and diagnosis of PTSS using risk ratio (dichotomous).

Preparing for eligibility screening

Before screening against eligibility criteria commences, search results retrieved from the outlined electronic databases will be assembled into a digital library and categorised by the search database using the reference management software EndNote (version X20, Clarivate Analytics, Philadelphia, PA). Any duplicate articles will be identified and eliminated at this stage.

Study selection

In the first reviewing round, two reviewers (FJ, DE) will independently screen titles and abstracts of articles within the digital library that potentially meet the pre-determined inclusion criteria. Both reviewers will then independently select potential eligible articles for full text screening and apply eligibility criteria to select appropriate articles for inclusion in the

For peer review only - http://bmjopen.bmj.com/site/about/guidelines.xhtml

review. Any disagreement will be resolved by consensus. If a resolution is not reached, a third reviewer (DF) will arbitrate any disagreement over study eligibility and resolve through discussion. Table 1 shows a checklist based on study eligibility criteria to ensure that studies are correctly identified and classified appropriately. A (PRISMA-P) flowchart will be generated and will document the selection process (inclusion and exclusion) along with reasons for exclusions.

Table 1: Eligibility criteria

**Study design**	▪ Randomised controlled trials<br>▪ Non-randomised controlled studies
**Study characteristics**	▪ Written in English language<br>▪ Study identified via electronic database search, grey literature, google scholar, or reference lists of eligible studies<br>▪ Full text article available<br>▪ Single case studies, case reports alongside any review articles, clinical guidelines, letters, editorials, studies with only abstracts and any other literature with no full text availability and articles not published in the English language will be excluded
**Participants**	▪ Adults aged (≥18 years) who have sustained a physically traumatic event resulting in one or more musculoskeletal injuries<br>▪ In studies with categorised aged group, >90% of participants must be adults (≥18 years)<br>▪ Studies focusing solely on patients with traumatic brain injury (TBI), spinal cord injuries, burns or deliberately self-injured patients
**Interventions**	▪ One or more non-pharmacological, non-surgical interventions (e.g. psychological interventions, exercise, eye-movements, and/or manual therapy), commencing within 3 months after a physically traumatic event that caused musculoskeletal injuries<br>▪ One or more comparator group
**Outcome measures**	▪ PTSS measured using one or more validated instruments/measures<br>▪ Medical records to obtain participants' clinical diagnoses of PTSS

For peer review only - http://bmjopen.bmj.com/site/about/guidelines.xhtml

Patient and public involvement

No patients or members of the public were directly involved in the design, writing or editing of this systematic review protocol. We will present the results of this review to our established patient and public involvement group.

Data extraction (selection and coding)

Data will be managed using EndNote, version 20 software (Clarivate Analytics, Philadelphia, PA). This will enable reviewers to access and share libraries, remove duplicates, review eligibility and store full texts and abstracts. Two reviewers will extract data from the included studies independently. Any disagreement over the eligibility of a study will be resolved through discussions and involve a third reviewer if needed. For missing data, up to two attempts will be made to contact study authors by email and/or phone to obtain further information. Data to be extracted will include: title of study, first author, year and country of publication, study sample size, demographic characteristics of the study sample (e.g. age, gender, ethnicity, marital status, and education level), description of the intervention(s), description of the comparator/control group(s), duration of the study, diagnostic tool(s) used to assess PTSS, intervention effect estimates on PTSS, proportion of eligible patients who agreed to participate, descriptors of compliance with the intervention, and PTSS outcome measure status (primary or secondary). Two reviewers will independently conduct data extraction from each study using a pre-defined data extraction sheet. Extracted outcome data will be pre- and post-intervention mean and standard deviation (SD) or risk ratio. Data presented as medians or alternate measures of spread will be converted to mean and SD. When only figures are presented (rather than numerical data within text), data will be extracted and analysed where possible using a software tool such as Web Plot Digitizer[54].

For peer review only - http://bmjopen.bmj.com/site/about/guidelines.xhtml

Risk of bias (quality) assessment

The Cochrane Risk of Bias tool V2 (RoB 2)[55] will be used to assess the risk of bias of each of the included randomised trials. This tool measures the potential risk of bias across five domains; (1) randomisation; (2) deviations from intended interventions; (3) missing outcome data; (4) measurement of the outcome data; and (5) reporting bias (selective outcome reporting). The Risk Of Bias in Non-randomised Studies of Interventions (ROBINS-I)[56] tool will be used to assess the risk of bias of non-randomized studies of interventions (NRSIs). Two reviewers (FJ, DE) will be involved in the quality assessment and any disagreements will be resolved through a third reviewer (DF). The Grading of Recommendations Assessment, Development, and Evaluation (GRADE)[57] approach will be used to assess the quality of the pooled evidence. Bias will be assessed as high, uncertain, or low risk. The risk of bias judgements will be taken into account in the final consideration of treatment effect, and studies with a high risk of bias will be flagged.

Strategy for data synthesis

Meta-analyses on reported outcomes will be performed depending upon the extent of between-study variation in participant populations, interventions, study design outcomes and methodological rigour and the number of studies reporting similar effect measures using the same assessment measures. A pairwise random-effects meta-analysis will be conducted depending upon the effect measures reported in the studies and similarities between individual studies, interventions and outcomes and the statistical heterogeneity, the assessment of whether genuine differences exist between results is low[58]. Heterogeneity will be evaluated using the I^2 statistical analysis: an I^2 of 50% and above is considered a high level of statistical heterogeneity[59]. Meta-analysis will be performed if heterogeneity between the studies is low (I^2<50%). Where possible, Standardised Mean Difference (SMD) and 95% CI will be extracted and reported as effect estimates of scales assessing PTSS. Additionally, Cohen's *d* will be

For peer review only - http://bmjopen.bmj.com/site/about/guidelines.xhtml

extracted or calculated: effect size will be defined as small (0.0-0.2), medium (0.3-0.7) and large (>0.8). Dichotomous outcomes (i.e. presence of PTSS or not) will be pooled separately and risk ratios with accompanying 95% CI extracted or calculated from raw data in exposed and comparator groups. If the level of heterogeneity and risk of bias is high between studies and pooled analysis of the studies is not possible, a narrative summary of the outcome of the selected studies will be undertaken and presented in the final review. Publication bias and small study effects (funnel plot asymmetry) will be assessed by the regression-based tests proposed by Debray and colleagues[60]. All analyses will be conducted in Stata 17.0 (Stata Corp, College Station, TX, USA).

Heterogeneity assessment

Assessment of the between-study heterogeneity will be statistically explored by univariate and multivariate meta-regression analyses, statistical significance will be set at ($p<0.05$). Differences in study characteristics (methodological diversity) and study populations (clinical diversity) will be further examined to explore sources of heterogeneity. Statistically significant predictors from the univariate analysis will be included in a multivariate meta-regression model, and the statistical significance will be determined at $p < 0.05$. Meta-regression will be performed in STATA using the 'metareg' command[61].

Sub-group analyses

Sub-group analyses will be performed depending on the number of studies identified. Sub-group analyses will be performed to consider the following; [1] type of intervention; [2] type of traumatic event. Sub-group analyses may be performed to explore trauma-focused CBT (TF-CBT), EMDR intervention or other active treatment conditions (e.g. exercise or mindfulness-based interventions). The level of heterogeneity between studies will be examined using the Cochrane Q-test and quantified by I^2 statistical test with 95% CI. Depending on the level of heterogeneity and study characteristics, both fixed and random effect models will be

For peer review only - http://bmjopen.bmj.com/site/about/guidelines.xhtml

used as summary effect estimates. The Mantel- Haenszel method[62] will be performed for fixed effect model if tests between study heterogeneity is not found. On the other hand, the DerSimonian and Laird method[63] will be used for random effects modelling if between-study heterogeneity is anticipated because of variations in clinical measurements (measures assessing psychological intervention), study population, type of traumatic event or regions across studies. This method incorporates a measure of the heterogeneity between studies. A minimum of two studies are generally considered sufficient to perform a meta-analysis[64].

Sensitivity analysis

If sufficient numbers of studies are available, we will conduct sensitivity analyses to assess the methodological quality and potential sources of heterogeneity of the included studies. Sources of variations may include assessment of psychological interventions, sampling strategy, adequate response and traumatic event. These will be stratified and separate sensitivity analyses conducted. A further analysis will be conducted excluding any studies with high risk of bias. We will explore the effect of restricting to studies with a low overall risk of bias.

Narrative synthesis

If the level of heterogeneity is high between studies and a meta-analysis is not feasible, we will carry out a narrative summary of the outcome of the selected studies and examined in more detail and presented in the final review outlining the reasons for the results reported in each study.

Publication bias and overall quality of the evidence

Presence of publication and small sample bias will be examined using the Egger test and inverted funnel plot technique[65]. The trim and fill method will be used to examine the magnitude of publication bias and estimate the overall effect of the remaining studies once

For peer review only - http://bmjopen.bmj.com/site/about/guidelines.xhtml

For peer review only

studies which cause the funnel asymmetry are removed and then missing effects will be imputed until the funnel plot is symmetrical[66]. The STATA command 'metatrim' will be used to perform the non-parametric trim and fill technique[67].

Certainty of evidence

The GRADE framework[57] will be used to assess the certainty of evidence for the outcome of interest across studies. Consistent with the GRADE approach, the quality of the certainty of evidence will be assessed as high, low moderate and very low. According to the GRADE five domains, we will assess imprecision, inconsistency, indirectness, risk of bias including publication bias. Applicability of the results based on the study population will also be rated when making judgement about the quality of evidence presented in the included studies[68]. The minimum number of included studies recommended when examining publication bias is ten[69].

DISCUSSION

The relative strengths and weaknesses identified in the included studies will be presented in the review. One of the strengths of the proposed study is to apply a reproducible and transparent procedure to comprehensively explore the effect of early non-pharmacological intervention on PTSS, delivered within three months of a traumatic event that caused musculoskeletal injuries. The findings of this review will provide estimates for the effectiveness of evaluated interventions in terms of improvement in or reduction of PTSS severity. Another strength will include an in-depth search strategy structured around the main concepts being examined in this review, and adapted for each search database, and robust quality appraisal of research-based evidence and heterogeneity assessment to evaluate studies included in this review. Some potential weaknesses are likely to include between-study heterogeneity in terms of PTSS diagnostic methods, descriptions of traumatic event, setting and/or country of study, publication bias and different non-pharmacological interventions (e.g.

For peer review only - http://bmjopen.bmj.com/site/about/guidelines.xhtml

For peer review only

psychoeducation, cognitive behavioural therapy, exercise, manual therapy, acupuncture, and eye movement desensitisation and reprocessing). Furthermore, PTSS commonly occurs with comorbidities and we will describe these, where available, in our review. This review will only include studies in English language, being recognised as a limitation of this review. A narrative summary of the study findings will be presented to overcome these issues if the results cannot be pooled.

Implications of results

This systematic review will provide a synthesis of the available literature exploring the effect of early non-pharmacological, non-surgical intervention on PTSS in adults (aged ≥18 years) who have experienced a traumatic event that caused musculoskeletal injuries. Findings of this review have the potential to assist clinicians to decide which interventions, either alone or in combination, have the greatest effect on PTSS when delivered within three months of a physically traumatic event, and may provide an opportunity for designing or tailoring future treatment for patients experiencing PTSS.

Ethics and dissemination

This review does not require ethical approval as only existing published data, available in scientific databases, will be used. Findings of this systematic review will be published in peer reviewed journals and presented at conferences. Any data generated from this systematic review will be made available from the corresponding author on reasonable request.

Author Contributions

FJ, DE and DF contributed equally to the conception of this protocol. FJ conceived the study design and drafted the first version of the protocol and was reviewed/revised by DE and DF. The final version was drafted by FJ. The search strategy was developed by FJ and iteration discussed with DE and DF. The final version was approved by DE and DF. The search will be

For peer review only

For peer review only - http://bmjopen.bmj.com/site/about/guidelines.xhtml

performed by FJ. FJ and DE will perform initial screening for study selection. FJ and DE will collect data from the included studies and conduct quality assessment. FJ and DE will perform data analysis/synthesis. DF will ensure data extraction consistency. FJ, DE and DF drafted and critically reviewed the manuscript and approved the final version. DF is guarantor.

Funding Statement

This project is funded by the National Institute for Health Research (NIHR) Surgical Reconstruction and Microbiology Research Centre (SRMRC) awarded to Deborah Falla. SRMRC is a research infrastructure for trauma research jointly funded by the NIHR, and the Ministry of Defence. The views expressed are those of the author(s) and not necessarily those of the NIHR or the Department of Health and Social Care.

Grant Number: No grant number

Competing Interests Statement

The authors have no competing interests.

Patient consent for publication

Not required.

Provenance and peer review

Not commissioned; externally peer reviewed.

For peer review only

REFERENCES

1. Rees S, Silove D, Chey T et al. Lifetime prevalence of gender-based violence in women and the relationship with mental disorders and psychosocial function. *JAMA*. 2011; 306:513–21.

2. O'Donnell ML, Creamer M, Pattison P, Atkin C. Psychiatric morbidity following injury. *Am J Psychiatry.* 2004; 161:507–14.

3. Lancaster CL, Teeter JB, Gros DF, Back SE. Post-traumatic stress disorder: Overview of evidence based assessment and treatment. *J Clin Med.* 2016; 5: 105.

4. Shalev AY, Gevonden M, Ratanatharathorn A et al. Estimating the risk of PTSD in recent trauma survivors: Results of the international consortium to predict PTSD (ICPP). *World J Psychiatry*. 2019; 18:77–87.

5. Kavan MG, Elsasser GN, Barone E. The Physician role in managing acute stress disorder. *Am Fam Physician*. 2012; 86:643-649.

6. Sareen J. Posttraumatic Stress Disorder in Adults: Impact, Comorbidity, Risk Factors, and Treatment. *Can J Psychiatry*. 2014; 59: 460–467.

7. American Psychiatric Association. (2013). Diagnostic and Statistical Manual of Mental Disorders. (5th ed). American Psychiatric Association; Arlington, VA, USA. Retrieved from: https://psychiatry.org/psychiatrists/practice/dsm

8. Lee C, Porter KM. Prehospital management of lower limb fractures. *Emerg Med J*. 2005; 22:660–3.

9. Accidents and injuries statistics: Causes of death - deaths by country of residence and occurrence. Eurostat; 2017. Available: https://ec.europa.eu/eurostat/databrowser/view/hlth_cd_aro/default/table?lang=en

10. Global status report on road safety 2018. Geneva: World Health Organization; 2018. Available: https://www.who.int/violence_injury_prevention/road_safety_status/2018/en/

11. National Audit Office Major Trauma Care in England 2010. London: The Stationery Office. Available: https://www.nao.org.uk/report/major-trauma-care-in-england/

12. Herron J, Hutchinson R, Lecky F, Bouamra O, Edwards A, Woodford M, et al. The impact of age on major orthopaedic trauma: an analysis of the United Kingdom trauma audit research network database. *Bone Joint J.* 2017; 99-B: 1677–80.

13. Cook A, Osler T, Glance L, Lecky F, Bouamra O, Weddle J, et al. Comparison of two prognostic models in trauma outcome. *Br J Surg*. 2018; 105:513–9.

For peer review only - http://bmjopen.bmj.com/site/about/guidelines.xhtml

14. Trauma and Audit Research Network. Modelling Trauma Workload: A Project for the Department of Health, 2009. Available: https://www.nao.org.uk/wp-content/uploads/2010/02/0910213.pdf

15. Davenport RA, Tai N, West A, et al. A major trauma centre is a specialty hospital not a hospital of specialties. *Br J Surg*. 2010; 97:109–117.

16. Sparks SW. Posttraumatic Stress Syndrome: What Is It?, *J Trauma Nurs*. 2018; 25:60-65.

17. Naylor JC, Dolber TR, Strauss JL, Kilts JD, Strauman TJ, Bradford DW, et al. A pilot randomized controlled trial with paroxetine for subthreshold PTSD in Operation Enduring Freedom/Operation Iraqi Freedom era veterans. *Psychiatry Res*. 2013; 206:318–320.

18. Greenburg N, Brooks S, Dunn R. Latest developments in post-traumatic stress disorder: diagnosis and treatment. *Br Med Bull*. 2015; 114: 147-155.

19. National Institute of Mental Health. Post-Traumatic Stress Disorder, 2020. Available: https://www.nimh.nih.gov/health/publications/post-traumatic-stress-disorder-ptsd

20. Royal College of Psychiatrists. Coping after a traumatic event, 2021. https://www.rcpsych.ac.uk/mental-health/problems-disorders/coping-after-a-traumatic-event

21. Alhussaini NW, Riaz M. Effectiveness of pharmacological and psychological interventions for treating post-traumatic stress disorder in adults with childhood abuse: protocol for a systematic review and network meta-analysis. *BMJOpen*. 2021; 11:e048790.

22. Fetzner MG, Asmundson GJG. Aerobic Exercise Reduces Symptoms of Posttraumatic Stress Disorder: A Randomized Controlled Trial. *Cogn Behav Ther*. 2015; 44:301-13.

23. Cloitre M, Courtois CA, Charuvastra A, Carapezza R, Stolbach BC, Green BL. Treatment of complex PTSD: Results of the ISTSS expert clinician survey on best practices. *J Trauma Stress*. 2011; 24:615–627.

24. Voorendonk EM, Sanches, SA, Tollenaar MS, De Jongh A, Van Minnen A.Augmenting PTSD treatment with physical activity: study protocol of the APPART study (Augmentation for PTSD with Physical Activity in a Randomized Trial). *Eur J Psychotraumatol*. 2022; 13:2016219.

25. Rosenow M, Munk N. Massage for Combat Injuries in Veteran with Undisclosed PTSD: a Retrospective Case Report. *Int J Ther Massage Bodywork*. 2021; 14: 4-11.

For peer review only - http://bmjopen.bmj.com/site/about/guidelines.xhtml

26. King HC, Spence DL, Hickey AH, Sargent P, Elesh R, Connelly CD.Auricular Acupuncture for Sleep Disturbance in Veterans With Post-Traumatic Stress Disorder: A Feasibility Study. *Mil Med*. 2015; 180:582-590.

27. Jonas DE, Cusack K, Forneris CA et al. Psychological and pharmacological treatments for adults with posttraumatic stress disorder (PTSD). AHRQ comparative effectiveness reviews. Rockville (MD: Agency for Healthcare Research and Quality, 2013. www.effectivehealthcare.ahrq.gov/reports/final.cfm.

28. Bisson JI, Shepherd JP, Joy D. Early cognitive-behavioural therapy for post-traumatic stress symptoms after physical injury. Randomised controlled trial. *Br J Psychiatry*. 2004;184:63-9.

29. Kitchiner NJ, Lewis C, Roberts NP. Active duty and ex-serving military personnel with post-traumatic stress disorder treated with psychological therapies: systematic review and meta-analysis. *Eur J Psychotraumatol*. 2019; 8: 1684226

30. Anish EJ. Exercise and its effects on the central nervous system. *Curr Sports Med Rep*. 2005; 4:18-23.

31. Tikac G, Unal A, Altug F. Regular exercise improves the levels of self-efficacy, self-esteem and body awareness of young adults. *J Sports Med Phys Fitness*. 2022; 62:157-161.

32. Butler AC, Chapman JE, Forman EM, Beck AT. The empirical status of cognitive-behavioral therapy: a review of meta-analyses. *Clin Psychol Rev*. 2006;26:17–31.

33. Jain S, Shapiro SL, Swanick S, Roesch SC, Mills PJ, Bell I, et al. A randomized controlled trial of mindfulness meditation versus relaxation training: effects on distress, positive states of mind, rumination, and distraction. *Ann Behav Med*. 2007;33:11–21.

34. Househam AM, Peterson CT, Mills PJ, Chopra D. The effects of stress and meditation on the immune system, human microbiota, and epigenetics. *Adv Mind Body Med*. 2017;31:10–25.

35. Hulett JM, Armer JM. A systematic review of spiritually based interventions and psychoneuroimmunological outcomes in breast cancer survivorship. *Integr Cancer Ther*. 2016;15:405–23.

36. Rosenbaum S, Vancampfort D, Steel ZJ, Ward, PB, Stubbs B. Physical activity in the treatment of post-traumatic stress disorder: A systematic review and meta-analysis. *Psychiatry Res*. 2015; 230: 130-136.

37. Shamseer L, Moher D, Clarke M. PRISMA-P Group. Preferred reporting for systematic review and meta-analysis protocols (PRISMA-P). BMJ 2015; 349: g7647.

For peer review only - http://bmjopen.bmj.com/site/about/guidelines.xhtml

38. McKenzie JE & Brennan SE. Chapter 12: Synthesizing and presenting findings using other methods. In: Higgins JPT, Thomas J, Chandler J, Cumpston M, Li T, Page MJ, Welch VA (editors). Cochrane Handbook for Systematic Reviews of Interventions version 6.2 (updated February 2021). Cochrane, 2021.

39. Busch FN, Milrod BL: Trauma-focused psychodynamic psychotherapy. *Psychiatr Clin North Am*. 2018; 41:277–287.

40. Donker T, Griffiths KM, Cuijpers P et al. Psychoeducation for depression, anxiety and psychological distress: a meta-analysis. *BMC Med*. 2009; 16:7-79.

41. Moloud R, Saeed Y, Mahmonir H et al. Cognitive-behavioral group therapy in major depressive disorder with focus on self-esteem and optimism: an interventional study. *BMC Psychiatry*. 2022; 28:299.

42. Shapiro, F. The Role of Eye Movement Desensitization and Reprocessing (EMDR) Therapy in Medicine: Addressing the Psychological and Physical Symptoms Stemming from Adverse Life Experiences. *Pem J*. 2014; 18:71-77.

43. Hegberg NJ, Hayes JP, Hayes SM. Exercise Intervention in PTSD: A Narrative Review and Rationale for Implementation. *Front Psychiatry*. 2019; 10: 133.

44. Bialosky JE, Bishop MD, Price DD, Robinson ME, George SZ. The Mechanisms of Manual Therapy in the Treatment of Musculoskeletal Pain: A Comprehensive Model. *Man. Ther*. 2009; 14:531-538.

45. Hollifield M. Acupuncture for Posttraumatic Stress Disorder: Conceptual, Clinical, and Biological Data Support Further Research. *CNS Neurosci Ther*. 2011; 17: 769–779.

46. Roland M, Morris R. A study of the natural-history of low-back-pain 2. Development of guidelines for trials of treatment in primary care. *Spine.* 1983; 8:145–50.

47. Thoresen S, Tambs K, Hussain A, Heir T, Johansen VA, Bisson JI. Brief measure of posttraumatic stress reactions: impact of Event Scale-6. *Soc Psychiatry Psychiatr Epidemiol.* 2010; 45:405-12.

48. Blake DD, Weathers F, Nagy L, et al. The development of a clinician-administered PTSD scale. *J Trauma Stress.* 1995; 8:75–90.

49. Blanchard EB, Jones-Alexander J, Buckley TC et al. Psychometric properties of the PTSD Checklist (PCL). Behav Res Ther 1996; 34: 669-673

50. Wilkins KC, Lang AJ, Norman SB. Synthesis of the Psychometric Properties of the PTSD Checklist (PCL) Military, Civilian, and Specific Versions. *Depress Anxiety*. 2011; 28: 596–606.

For peer review only - http://bmjopen.bmj.com/site/about/guidelines.xhtml

51. Weathers FW, Litz BT, Keane TM, et al. (2014, February 5). PTSD Checklist for DSM-5 (PCL-5). National Center for PTSD; 2014. Available: http://www.ptsd.va.gov/professional/assessment/adult-sr/ptsd-checklist.asp.

52. Foa EB, McLean CP, Zang Y et al. Psychometric properties of the Posttraumatic Diagnostic Scale for DSM-5 (PDS-5). *Psychol Assess.* 2016; 28: 1166-1171.

53. Bryant RA, Moulds ML, Guthrie RM. Acute stress disorder scale: a self-report measure of acute stress disorder. *Psychol Assess.* 2000; 12:61-8.

54. Rohatgi A. WebPlotDigitizer - Extract data from plots, images, and maps. 2020. Available: https://automeris.io/WebPlotDigitizer/

55. Sterne AC, Savović J, Page MJ et al. RoB 2: a revised tool for assessing risk of bias in randomised trials. BMJ. 2019; 366: l4898.

56. Sterne, JAC, Hernán, MA, Reeves BC et al. ROBINS-I: a tool for assessing risk of bias in non-randomised studies of interventions. *BMJ.* 2016; 355:i4919.

57. Balshem H, Helfand M, Schünemann H.J et al. GRADE guidelines: 3. Rating the quality of evidence. *J. Clin. Epidemiol.* 2011; 64:401-40.

58. Higgins JPT, Thomas J, Chandler J, Cumpston M, Li T, Page MJ, Welch VA (editors). Cochrane Handbook for Systematic Reviews of Interventions version 6.3 (updated February 2022). Cochrane, 2022. Available: www.training.cochrane.org/handbook.

59. Higgins JP, Green S. Cochrane handbook for systematic reviews of interventions, 2011. Available: http://www.mri.gov.lk/assets/Uploads/Research/Cochrane-Hand-booktext.pdf.

60. Debray TPA, Moons KGM, Riley RD. Detecting small-study effects and funnel plot asymmetry in meta-analysis of survival data: A comparison of new and existing tests. *Res Synth Methods*. 2018: 9:41-50.

61. Harbord RM, Higgins JPT. Meta-regression in Stata. Stata Journal 2008; 8:493-419. Available: http://www.stata-journal.com/article.html?article=sbe23_1.

62. Mantel N, Haenszel W. Statistical aspects of the analysis of data from retrospective studies of disease. *J Natl Cancer Inst.* 1959; 22:719-48.

63. DerSimonian R, Laird N. Meta-analysis in clinical trials. *Control Clin Trials*. 1986; 7: 177-188.

64. Kang H. Statistical considerations in meta-analysis. *Hanyang Med Rev.* 2015;35:23–32

For peer review only - http://bmjopen.bmj.com/site/about/guidelines.xhtml

65. Egger M , Smith GD , Schneider M , et al . Bias in meta-analysis detected by a simple, graphical test. *BMJ*. 1997;315:629–34.

66. Duval S, Tweedie RL. Trim and fill: A simple funnel plot-based method of testing and adjusting for publication bias in meta-analysis. *Biometrics*. 2000b; 56:276-284.

67. Sterne JAC, Newton HJ, Cox NJ. Meta-Analysis in Stata: An Updated Collection from the Stata Journal. 2009; College Station, TX, Stata Press.

68. Guyatt G, Oxman AD, Akl EA, et al. GRADE guidelines: 1. Introduction-GRADE evidence profiles and summary of findings tables. *J Clin Epidemiol*. 2011; 64:383–94.

69. Sterne JA, Sutton AJ, Ioannidis JPA. Recommendations for examining and interpreting funnel plot asymmetry in meta-analyses of randomised controlled trials. *BMJ*. 2011; 343: d4002.

For peer review only

For peer review only - http://bmjopen.bmj.com/site/about/guidelines.xhtml

Supplementary file 1: PRISMA-P Checklist: This checklist has been adapted for use with protocol submissions to *Systematic Reviews* from Table 3 in Moher D et al: Preferred reporting items for systematic review and meta-analysis protocols (PRISMA-P) 2015 statement. *Systematic Reviews* 2015 **4**:1

Early interventions for post-traumatic stress following musculoskeletal trauma: Protocol for a systematic review and meta-analysis

Section/topic	#	Checklist item	Information reported: Yes	Information reported: No	Page number(s)
**ADMINISTRATIVE INFORMATION**					
**Title**					
Identification	1a	Identify the report as a protocol of a systematic review	☒	☐	1
Update	1b	If the protocol is for an update of a previous systematic review, identify as such	☐	☒	N/A
**Registration**	2	If registered, provide the name of the registry (e.g., PROSPERO) and registration number in the Abstract	☒	☐	3
**Authors**					
Contact	3a	Provide name, institutional affiliation, and e-mail address of all protocol authors; provide physical mailing address of corresponding author	☒	☐	1
Contributions	3b	Describe contributions of protocol authors and identify the guarantor of the review	☒	☐	17-18
**Amendments**	4	If the protocol represents an amendment of a previously completed or published protocol, identify as such and list changes; otherwise, state plan for documenting important protocol amendments	☐	☒	N/A
**Support**					
Sources	5a	Indicate sources of financial or other support for the review	☒	☐	18
Sponsor	5b	Provide name for the review funder and/or sponsor	☒	☐	18
Role of sponsor/funder	5c	Describe roles of funder(s), sponsor(s), and/or institution(s), if any, in developing the protocol	☒	☐	18
**INTRODUCTION**					
**Rationale**	6	Describe the rationale for the review in the context of what is already known	☒	☐	7
**Objectives**	7	Provide an explicit statement of the question(s) the review will address with reference to participants, interventions, comparators, and outcomes (PICO)	☒	☐	8-9
**METHODS**					
**Eligibility criteria**	8	Specify the study characteristics (e.g., PICO, study design, setting, time frame) and report characteristics (e.g., years considered, language, publication status) to be used as criteria for eligibility for the review	☒	☐	6-8
**Information sources**	9	Describe all intended information sources (e.g., electronic databases, contact with study authors, trial	☒	☐	8

For peer review only

BioMed Central The Open Access Publisher

Section/topic	#	Checklist item	Information reported Yes	Information reported No	Page number(s)
		registers, or other grey literature sources) with planned dates of coverage			
**Search strategy**	10	Present draft of search strategy to be used for at least one electronic database, including planned limits, such that it could be repeated	☒	☐	8
STUDY RECORDS					
Data management	11a	Describe the mechanism(s) that will be used to manage records and data throughout the review	☒	☐	8
Selection process	11b	State the process that will be used for selecting studies (e.g., two independent reviewers) through each phase of the review (i.e., screening, eligibility, and inclusion in meta-analysis)	☒	☐	8
Data collection process	11c	Describe planned method of extracting data from reports (e.g., piloting forms, done independently, in duplicate), any processes for obtaining and confirming data from investigators	☒	☐	12
**Data items**	12	List and define all variables for which data will be sought (e.g., PICO items, funding sources), any pre-planned data assumptions and simplifications	☒	☐	8-9
**Outcomes and prioritization**	13	List and define all outcomes for which data will be sought, including prioritization of main and additional outcomes, with rationale	☒	☐	9
**Risk of bias in individual studies**	14	Describe anticipated methods for assessing risk of bias of individual studies, including whether this will be done at the outcome or study level, or both; state how this information will be used in data synthesis	☒	☐	13
DATA					
**Synthesis**	15a	Describe criteria under which study data will be quantitatively synthesized	☒	☐	13-14
	15b	If data are appropriate for quantitative synthesis, describe planned summary measures, methods of handling data, and methods of combining data from studies, including any planned exploration of consistency (e.g., I^2, Kendall's tau)	☒	☐	10-11
	15c	Describe any proposed additional analyses (e.g., sensitivity or subgroup analyses, meta-regression)	☒	☐	13-14
	15d	If quantitative synthesis is not appropriate, describe the type of summary planned	☒	☐	15-16
**Meta-bias(es)**	16	Specify any planned assessment of meta-bias(es) (e.g., publication bias across studies, selective reporting within studies)	☒	☐	15-16
**Confidence in cumulative evidence**	17	Describe how the strength of the body of evidence will be assessed (e.g., GRADE)	☒	☐	16

For peer review only

BioMed Central
The Open Access Publisher

For peer review only - http://bmjopen.bmj.com/site/about/guidelines.xhtml

Early interventions for post-traumatic stress following musculoskeletal trauma: Protocol for a systematic review and meta-analysis

Medline search strategy

1. Post-traumatic stress symptom

Post-traumatic stress.mp **OR** Post-traumatic stress disorder.mp **OR** Post traumatic.mp **OR** Post-traumatic stress symptom.mp **OR** Stress symptoms.mp **OR** Post-trauma*.mp **OR** PTSS.mp or PTSD.mp **OR** Acute stress disorder.mp **OR** ASD.mp

2. Musculoskeletal trauma

Musculoskeletal trauma.mp **OR** musculoskeletal injury.mp **OR** Orthopaedic trauma.mp **OR** Orthopaedic injuries.mp **OR** Orthopedic trauma.mp **OR** Orthopedic injury.mp **OR** Musculoskeletal.mp **OR** Musculoskeletal inj* **OR** Lower extremity inj* **OR** Upper extremity inj* **OR** Exp fractures, bone **OR** Exp whiplash injuries **OR** Whiplash.mp **OR** Athletic injuries.mp **OR** Traumatic injuries.mp **OR** Falls.mp **OR** Traffic collision.mp **OR** Exp multiple trauma **OR** Traumatic inj* **OR** Gunshot injury* **OR** Stab wound*

3. Intervention

Psychotherapies.mp **OR** psychotherap* **OR** physiotherap* **OR** physical therap* **OR** emdr.mp **OR** eye movement desensiti* **OR** prevention.mp **OR** intervention.mp **OR** therap* **OR** psychoeducation.mp **OR** treatment.mp **OR** cognitive behavioural therapy.mp **OR** CBT.mp **OR** rehabilitation.mp **OR** imagery.mp **OR** exercis*.mp **OR** movement therapy.mp **OR** exercise training.mp **OR** physical activit*.mp **OR** aerobic exercise.mp **OR** physical exercise.mp **OR** resistance training.mp **OR** strength training.mp **OR** endurance exercise.mp **OR** physical therap*.mp **OR** manual therap*.mp **OR** massage.mp **OR** acupuncture **OR** aerobic.mp

For peer review only - http://bmjopen.bmj.com/site/about/guidelines.xhtml

CINAHL search strategy

1. Post-traumatic stress symptom

Post traumatic **OR** Post-traumatic stress symptom **OR** Post-trauma **OR** PTSS **OR** Post-traumatic stress **OR** Post-traumatic stress disorder **OR** Post-traumatic stress symptom **OR** PTSD **OR** Acute stress disorder **OR** ASD

2. Musculoskeletal trauma

Musculoskeletal trauma **OR** Musculoskeletal injury **OR** Orthopaedic trauma **OR** Orthopaedic injury **OR** Orthopedic trauma **OR** Lower extremity injury **OR** Upper extremity injury **OR** Fractures, bone **OR** Whiplash injuries **OR** Whiplash **OR** Athletic injury **OR** Multiple trauma **OR** Traumatic injury **OR** Traumatic injuries **OR** Falls **OR** Gunshot injury **OR** Stab wound **OR** Traffic collision

3. Intervention

Psychotherapies **OR** psychotherapy **OR** physiotherapy **OR** physical therapy **OR** EMDR **OR** eye movement desensitisation **OR** prevention **OR** intervention **OR** therapy **OR** psychoeducation **OR** treatment **OR** cognitive behavioural therapy **OR** CBT **OR** rehabilitation **OR** imagery **OR** exercise **OR** movement therapy **OR** exercise training **OR** physical activity **OR** aerobic exercise **OR** physical exercise **OR** resistance training **OR** strength training OR endurance exercise **OR** physical therapy **OR** manual therapy **OR** massage **OR** acupuncture **OR** aerobic

For peer review only

EMBASE search strategy

1. Post-traumatic stress symptom

Post traumatic.ab, ti **OR** Post-traumatic stress symptom$.ab, ti **OR** Post-trauma.mp **OR** PTSS.mp **OR** Post traumatic stress.mp **OR** Post-traumatic stress disorder.mp **OR** Post traumatic.mp **OR** Post-traumatic stress symptom.mp **OR** PTSD.mp **OR** Acute stress disorder.mp **OR** ASD.mp

PTSS.tw

2. Musculoskeletal trauma

Musculoskeletal trauma.mp **OR** musculoskeletal injury.mp **OR** Orthopaedic trauma.mp **OR** orthopedic trauma.mp **OR** Fractures, bone.mp **OR** Whiplash injuries.mp **OR** Whiplash.mp **OR** Multiple trauma.mp **OR** Traumatic injuries.mp **OR** Falls.mp **OR** Gunshot injury.mp **OR** Stab wound.mp **OR** Traffic collision.mp **OR** Musculoskeletal inj$.ab,ti **OR** Orthopaedic inj$.ab,ti **OR** Orthopedic inj$.ab,ti **OR** Lower extremity inj$.ab,ti **OR** Upper extremity inj$.ab,ti **OR** Athletic inj$.ab,ti **OR** Traumatic inj$.ab,ti

3. Intervention

Psychotherap$.ab,ti **OR** Psychotherapies.mp **OR** Psychotherapy.mp **OR** physiotherapy$.ab,ti **OR** Physiotherapies.mp **OR** Physiotherapy.mp **OR** physical therap$.ti,ab **OR** physical therapies.mp **OR** physical therapy.mp **OR** EMDR.mp **OR** eye movement desensitisation.mp **OR** desensitization.mp **OR** prevention.mp **OR** intervention.mp **OR** therap$.ab,ti **OR** therapies.mp **OR** therapy.mp **OR** psychoeducation.mp **OR** treatment.mp **OR** cognitive behavioural therapy.mp **OR** CBT.mp **OR** rehabilitation.mp **OR** imagery.mp OR exercise.mp **OR** movement therapy.mp **OR** exercise training.mp **OR** physical activity.mp **OR** aerobic exercise.mp **OR** physical exercise.mp **OR** resistance training.mp **OR** strength training.mp **OR** endurance exercise.mp

For peer review only - http://bmjopen.bmj.com/site/about/guidelines.xhtml

OR physical therapy.mp **OR** manual therapy.mp **OR** massage.mp **OR** acupuncture **OR** aerobic.mp

PsycINFO search strategy

1. Post-traumatic stress symptom

Post-traumatic stress disorder.mp **OR** post-traumatic stress disorders.mp **OR** posttraumatic stress disorder.mp **OR** posttraumatic stress disorders.mp **OR** disorder* **OR** post-traumatic.mp **OR** post-traumatic stress symptom*.mp **OR** post traumatic stress symptom*.mp **OR** PTSS.mp **OR** PTSD.mp **OR** acute stress disorder.mp **OR** ASD.mp

2. Musculoskeletal trauma

Musculoskeletal trauma.mp **OR** musculoskeletal injur*.mp **OR** orthopaedic trauma.mp **OR** orthopaedic injur*.mp **OR** orthopedic trauma.mp **OR** lower extremity injur*.mp **OR** upper extremity injur*.mp **OR** fracture*.mp **OR** bone.mp **OR** whiplash injur*.mp **OR** whiplash **OR** athletic injur*.mp **OR** multiple trauma.mp **OR** traumatic injur*.mp **OR** traumatic injur*.mp **OR** falls.mp **OR** gunshot injur*.mp **OR** stab wound*.mp **OR** traffic collision*.mp

3. Intervention

Psychotherap* **OR** Psychotherapies.mp **OR** Psychotherapy.mp **OR** physiotherapy* **OR** Physiotherapies.mp **OR** Physiotherapy.mp **OR** physical therap* **OR** physical therapies.mp **OR** physical therapy.mp **OR** EMDR.mp **OR** eye movement desensitisation.mp **OR** desensitization.mp **OR** prevention.mp **OR** intervention.mp **OR** therap* **OR** therapies.mp **OR** therapy.mp **OR** psychoeducation.mp **OR** treatment.mp **OR** cognitive behavioural therapy.mp **OR** CBT.mp **OR** rehabilitation.mp **OR** imagery.mp OR exercis* OR movement therapy.mp OR exercise training.mp OR physical activit* OR aerobic exercise.mp OR physical exercise.mp OR resistance training.mp OR strength training.mp OR endurance exercise.mp **OR** physical therap* OR manual therap* OR massage.mp OR acupuncture OR aerobic.mp

For peer review only - http://bmjopen.bmj.com/site/about/guidelines.xhtml

PubMed search strategy

1. Post-traumatic stress symptom

Post-traumatic stress **OR** Post-traumatic stress disorder **OR** Post traumatic **OR** Post-traumatic stress symptom **OR** Stress symptoms **OR** Post-trauma **OR** PTSS **OR** PTSD **OR** Acute stress disorder **OR** ASD

2. Musculoskeletal trauma

Musculoskeletal trauma **OR** musculoskeletal injury **OR** Orthopaedic trauma **OR** Orthopaedic injuries **OR** Orthopedic trauma **OR** Orthopedic injury **OR** Musculoskeletal **OR** Orthopaedic **OR** Musculoskeletal injury **OR** Lower extremity injury **OR** Upper extremity injury **OR** fractures **OR** bone **OR** whiplash injuries **OR** Whiplash **OR** Athletic injuries **OR** Traumatic injuries **OR** Falls **OR** Traffic collision **OR** multiple trauma **OR** Traumatic injury **OR** Gunshot injury OR Stab wound

3. Intervention

Psychotherapy **OR** Psychotherapies **OR** Physiotherapy **OR** Physiotherapies **OR** physical therapy **OR** physical therapies **OR** EMDR **OR** eye movement desensitisation **OR** desensitization **OR** prevention **OR** intervention **OR** therapy **OR** therapies **OR** psychoeducation **OR** treatment **OR** cognitive behavioural therapy **OR** CBT **OR** rehabilitation **OR** imagery **OR** exercise **OR** movement therapy **OR** exercise training **OR** physical activity **OR** aerobic exercise **OR** physical exercise **OR** resistance training **OR** strength training OR endurance exercise **OR** physical therapy **OR** manual therapy **OR** massage **OR** acupuncture **OR** aerobic

For peer review only - http://bmjopen.bmj.com/site/about/guidelines.xhtml

Web of Science search strategy

1. Part 1: Post-traumatic stress symptom

Post-traumatic stress **OR** Post-traumatic stress disorder **OR** Post traumatic **OR** Post-traumatic stress symptom **OR** Stress symptoms **OR** Post-trauma **OR** PTSS **OR** PTSD **OR** Acute stress disorder **OR** ASD

2. Musculoskeletal trauma

Musculoskeletal trauma **OR** musculoskeletal injury **OR** Orthopaedic trauma **OR** Orthopaedic injuries **OR** Orthopedic trauma **OR** Orthopedic injury **OR** Musculoskeletal **OR** Orthopaedic **OR** Musculoskeletal injury **OR** Lower extremity injury **OR** Upper extremity injury **OR** fractures **OR** bone **OR** whiplash injuries **OR** Whiplash **OR** Athletic injuries **OR** Traumatic injuries **OR** Falls **OR** Traffic collision **OR** multiple trauma **OR** Traumatic injury **OR** Gunshot injury OR Stab wound

3. Intervention

Psychotherapy **OR** Psychotherapies **OR** Physiotherapy **OR** Physiotherapies **OR** physical therapy **OR** physical therapies **OR** EMDR **OR** eye movement desensitisation **OR** desensitization **OR** prevention **OR** intervention **OR** therapy **OR** therapies **OR** psychoeducation **OR** treatment **OR** cognitive behavioural therapy **OR** CBT **OR** rehabilitation **OR** imagery **OR** exercise **OR** movement therapy **OR** exercise training **OR** physical activity **OR** aerobic exercise **OR** physical exercise **OR** resistance training **OR** strength training **OR** endurance exercise **OR** physical therapy **OR** manual therapy **OR** massage **OR** acupuncture **OR** aerobic

For peer review only - http://bmjopen.bmj.com/site/about/guidelines.xhtml
